# Increased caecal *Intestinimonas* abundance inhibits *E. tenella* gametogenesis via *EtGFAT* regulation and alleviates infection through immunity

**DOI:** 10.1186/s40168-025-02302-8

**Published:** 2026-01-12

**Authors:** Jun-Yi Li, Hai-Bin Huang, Chun-Wei Shi, Tian-Xu Pan, Ming-Han Li, Nan Wang, Jia-Jin Shan, Yan-Long Jiang, Wen-Tao Yang, Xin Cao, Jian-Zhong Wang, Jia-Yao Guan, Shu-Yuan Yu, Chun-Feng Wang, Gui-Lian Yang

**Affiliations:** 1 College of Veterinary Medicine, Jilin Agricultural University, Changchun, 130118 China; 2 Jilin Provincial Engineering Research Center of Animal Probiotics, Jilin Provincial Key Laboratory of Animal Microecology and Healthy Breeding, Jilin Agricultural University, Changchun, 130118 China; 3 Engineering Research Center of Microecological Vaccines (Drugs) for Major Animal Diseases, Ministry of Education, Jilin Agricultural University, Changchun, 130118 China

**Keywords:** *Eimeria tenella*, *Intestinimonas*, Caecal microbiota, *EtGFAT*, CD8^+^ T cells, Gametogony

## Abstract

**Background:**

Chicken coccidiosis caused by *Eimeria tenella* (*E*. *tenella*) poses a major threat to global poultry production, with its tropism for the caecal microenvironment and dynamic interactions with the resident microbiota remaining incompletely understood. The caecal microbiota plays a critical role in host‒parasite interplay, yet the mechanisms through which microbial homeostasis influences *E*. *tenella* development and host resistance remain elusive. This study aimed to elucidate the causal relationship between caecal dysbiosis and *E*. *tenella* pathogenesis, with a focus on identifying microbiota-derived regulators of parasite development and host immunity.

**Results:**

Antibiotic-induced caecal dysbiosis (ABX) significantly impaired *E*. *tenella* macrogametogenesis, demonstrating microbiota-dependent regulation of parasitic development. Faecal microbiota transplantation (FMT) validated this causal link, revealing that microbial reconstitution restored parasite maturation. Notably, *Intestinimonas* spp. were identified as key inhibitors of *E*. *tenella* development through transcriptional regulation of the *EtGFAT* gene (*Eimeria tenella* glucosamine: fructose-6-phosphate aminotransferase), a critical mediator of macrogamete formation. Furthermore, the transplantation of *Intestinimonas butyriciproducens* (*I*. *butyriciproducens*) attenuated clinical manifestations of infection while increasing IFN-γ secretion from CD8^+^ T lymphocytes, thereby enhancing host resistance to *E*. *tenella.*

**Conclusions:**

This study revealed that caecal microbiota homeostasis is indispensable for *E*. *tenella* developmental progression and highlights *Intestinimonas* as a pivotal microbial regulator of parasite biology. The dual role of *I*. *butyriciproducens* in suppressing parasitic virulence and potentiating adaptive immune responses underscores the therapeutic potential of microbiota-targeted strategies. These findings provide a foundation for the development of novel anticoccidial interventions through targeted manipulation of caecal microbial communities.

Video Abstract

**Supplementary Information:**

The online version contains supplementary material available at 10.1186/s40168-025-02302-8.

## Background

Chicken coccidiosis, an intestinal protozoan disease predominantly caused by *Eimeria* species, poses a severe threat to global poultry production, resulting in substantial productivity losses and significant economic burdens [1]. Among the seven species that infect chickens, *E*. *tenella* is among the most virulent, exhibiting exclusive tropism for the caecum and causing severe haemorrhagic diarrhoea and elevated mortality [2, 3]. The currently employed control strategies rely heavily on anticoccidial drugs and live vaccines. However, the increasing issue of drug resistance [4] and the challenges associated with vaccine implementation underscore the urgent need for novel intervention strategies [5].

The caecal microbiota, a complex community dominated by Bacteroidetes, Firmicutes and Actinobacteria [6], plays a fundamental role in maintaining chicken health and productivity. As the primary niche for *E*. *tenella*, the caecum facilitates the dynamic interplay between the parasite and the resident microbiota [7]. *E*. *tenella* infection significantly remodels the microbial community, reducing the abundance of beneficial taxa such as *Lactobacillus* and *Bifidobacterium* [8] and inducing inflammation that compromises microbiota integrity [9]. Conversely, the microbiota is involved in host defence, with various probiotic formulations demonstrating potential in alleviating infection-induced gastrointestinal disturbances [10–12].

Despite these observations, a critical knowledge gap remains: the precise mechanisms by which the caecal microbiota influences *E*. *tenella* development and host‒parasite interactions are elusive. While alterations in microbial composition postinfection have been documented, whether microbial homeostasis is a prerequisite for the progression of *E*. *tenella* through its developmental stages, particularly through the complex sexual stages (gametogony) critical for transmission, is unknown. Furthermore, the identification of specific microbiota-derived regulators that can directly inhibit parasitic development or potentiate host immunity is lacking. The molecular interactions between microbial metabolites and key parasitic genes, especially those governing gametocyte formation and oocyst wall biogenesis (e.g. EtGAM56 [13]), remain entirely unexplored.

This study aimed to move beyond correlation and establish causality in the relationship between caecal microbiota homeostasis and *E*. *tenella* pathogenesis. We hypothesised that the microbiota is essential for parasite development and that its disruption could reveal key microbial taxa and mechanisms governing parasite biology. By employing an antibiotic-induced dysbiosis (ABX) model, faecal microbiota transplantation (FMT) and integrated multiomics approaches, we sought to first determine the causal role of the microbiota in* E*. *tenella* gametogony, then identify specific microbiota-derived inhibitors of parasite development, and ultimately elucidate the underlying mechanisms, focusing on both direct parasitic gene regulation and host immunity. Our findings provide novel insights into the microbiota‒parasite interplay and lay the groundwork for developing microbiota-based anticoccidial interventions.

## Methods

### Animals, parasites, bacteria and cell lines

Healthy 1-day-old male Arbor Acres broilers (AA broilers) were obtained from the Changchun hatchery in Jilin and reared in the animal husbandry facilities at the Research Base of Jilin Agricultural University. The environment was thoroughly disinfected to ensure that it was pathogen free, including free from coccidia. The feed was prepared in accordance with the People’s Republic of China (PRC) standards (GB/T 5916–2020), with AA broilers provided ad libitum access to water and feed. Lighting, temperature and other husbandry conditions were implemented as per the Broiler Management Handbook by Aviagen.

*E. tenella* was provided by the Jilin Provincial Key Laboratory of Animal Microecology and Healthy Breeding [14] and stored at 4 °C with 2.5% potassium dichromate (207,802; Sigma). The DF-1 cell line was obtained from the ATCC repository (CRL-3586™), while *I*.* butyriciproducens* was obtained from the DSMZ (DSM 26588, type strain).

### ABX treatment, FMT and infection

All the experimental animals were 1-day-old SPF AA broilers. ABX treatment consisted of vancomycin (5 g/L), neomycin (10 g/L), metronidazole (10 g/L) and amphotericin B (0.1 g/L), prepared in sterile water and administered orally at 10 mL/kg daily for 14 days, with 1 g/L penicillin added to the drinking water daily (CV11721, CN7741, CM7361, CA2021, CA2031, Coolaber).

#### FMT

Caecal contents from age-matched SPF broilers were collected and administered 10^9^ cfu per chick per day via enema tubes (2 mm × 10 cm) inserted into the rectum to replenish the caecum. The optical density at 600 nm (OD_600_) = 1 represented a dose of 2 × 10^9^ cfu/mL. The transplantation was conducted over five consecutive days. The uninfected negative control group (MOCK) underwent routine feeding and was noninfected, serving solely as the donor for FMT.

The conventional infection group (CON) was infected only with *E*. *tenella*. All infected groups were simultaneously inoculated with *E*. *tenella*-sporulated oocysts at a dose of 50,000 oocysts per chick. The detailed allocation of the animals and the experimental design are summarised in Table 1. Chickens were housed in pens with 6 birds per pen, and each pen was considered an independent experimental unit. This design ensured that all the chickens within a single pen were subjected to identical environmental and treatment conditions, minimising interpen variability and ensuring the statistical robustness of the data.

**Table 1:** Summary of animal usage

No	Experimental purpose	Group	Number of animals per group (*n*)	Location in manuscript
1	Oocysts collection and sporozoites purification	–	30	Figure 1
2	Merozoites purification	–	20	Figure 1
3	Caecal microbiota influences gametogony	MOCK, ABX, CON	30	Figures 1& 2
4	FMT and Key Bacterial Genus Screening	MOCK, ABX, CON, FMT	30	Figure 3
5	RNA-seq analysis of *E*. *tenella* gametes	ABX, CON, FMT	30	Figure 4
6	The effects of BC and CS on the Merozoites	–	20	Figure 5
7	Effect of IB on CD8^+^ T cells in the caecal tonsils at 7 dpi with *E*.* tenella*	MOCK, ABX, CON, FMT, BC, CS	10	Figure 6
8	IB regulates host immunity against *E*. *tenella* infection	MOCK, CON, ABX, FMT, IB, NaB	30	Figures 7 and 8

**Fig. 1:**
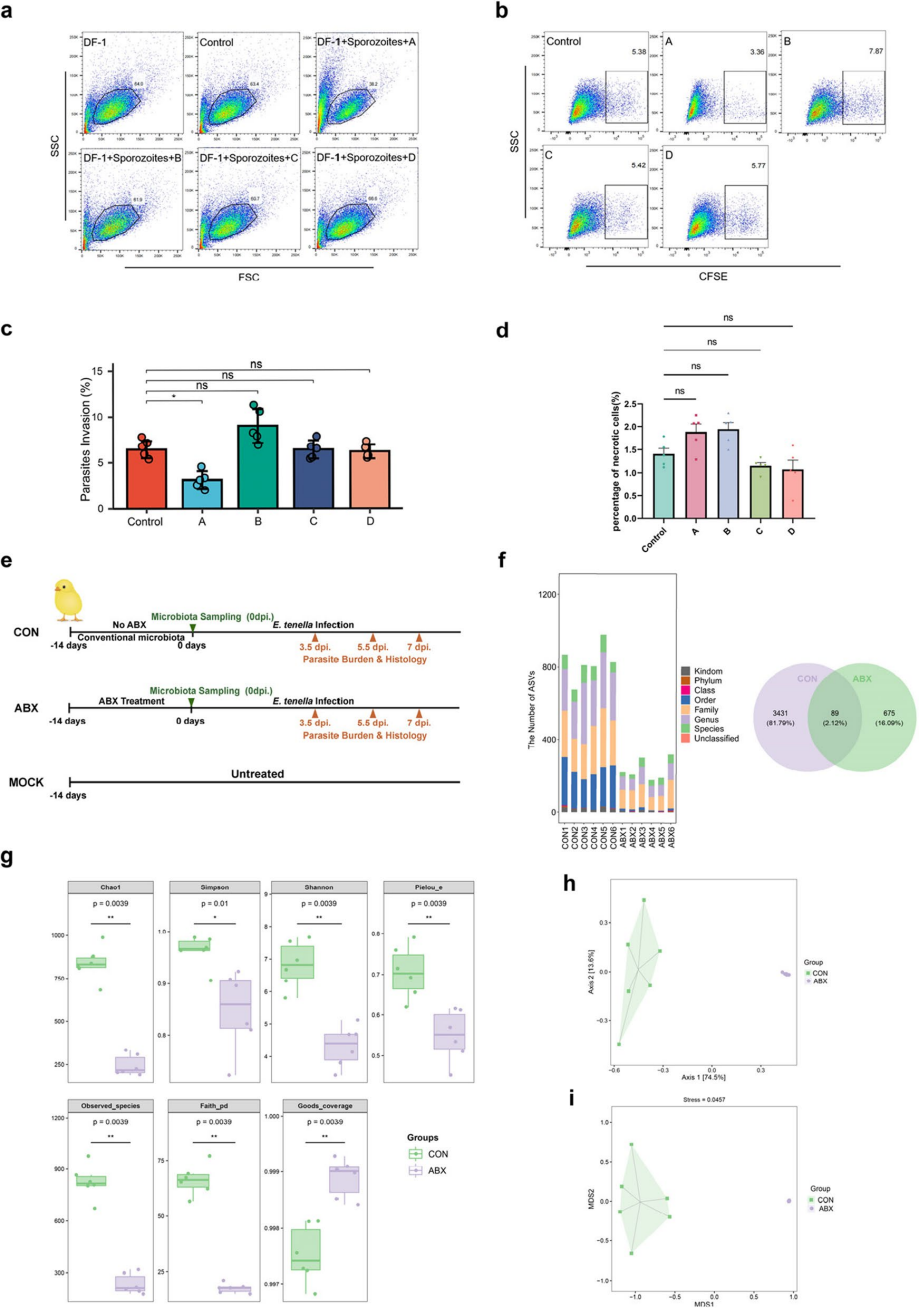
ABX induces significant dysbiosis in the chicken caecal microbiota without impairing the invasion efficiency of *E*. *tenella* sporozoites or the survival of merozoites. Flow cytometry analysis of *E*. *tenella* sporozoite invasion in DF-1 cells treated with various ABX concentrations. The left panel (**a**) shows the FSC-SSC scatter plot identifying cell populations, while the right panel (**b**) displays CFSE-labelled sporozoites in DF-1 cells treated with ABX concentrations: A (100%), B (10%), C (1%) and D (0.1%). CFSE-positive cells indicate sporozoite invasion. **c** Sporozoite invasion rates were quantified for CFSE-positive DF-1 cells in each ABX treatment group. No significant differences were detected among the groups (*p < 0.05). **d** Necrosis levels in merozoites were compared across experimental groups, with no statistically significant differences in viability observed. **e** One-day-old AA broilers were administered ABX for 14 days to induce caecal dysbiosis, followed by oral infection with 50,000 sporulated *E*. *tenella* oocysts. The experimental groups included MOCK (untreated, noninfected control), CON (infected control), and ABX (ABX-treated, infected). **f** Amplicon sequence variants (ASVs) in the ABX and CON groups were taxonomically annotated at 0 dpi. The bar chart depicts the hierarchical ASV distribution from the phylum to the species level, while the Venn diagram highlights shared and unique ASVs, illustrating the impact of ABX treatment on microbiota composition. **g** Alpha diversity analysis revealed significant reductions in caecal microbiota diversity within the ABX group. Marked declines were observed in the Chao1, Simpson, Shannon, Pielou, observed species, Faith’s PD, and Good’s coverage indices (Kruskal‒Wallis test; **p < 0.01, *p < 0.05). **h** PCoA plot based on Bray‒Curtis distances showing distinct β diversity between the ABX and CON groups, with clear separation (**p < 0.01). **i** The NMDS plot of β diversity further confirms the differences in microbiota composition, with distinct clustering between the ABX and CON groups (**p < 0.01)

### Evaluation and calculation of the anticoccidial index

The body weight of each chicken was recorded on the day of infection (14 days old) and at the end of the experiment (7 days post-infection, 21 days old). The average body weight gain (ABWG) for each group was calculated as follows: ABWG (g) = average body weight at 21 days − average body weight at 14 days. The relative body weight gain rate (RBWG, %) was calculated as follows: RBWG (%) = (ABWG of treated group/ABWG of non − infected MOCK group) × 100. The number of oocysts per gram of droppings (OPG) was calculated according to the McMaster counting technique [15] at the end of the challenge. The lesions on the caeca on day 7 post challenge were scored on a scale from 0 to 4 [16]. The anticoccidial index (ACI) is an artificial criterion for determining the anticoccidial effect and was calculated as follows: (relative rate of weight gain + survival rate) − (lesion value + oocyst value) [17]. An ACI > 180 indicated high performance, a 160 ≤ ACI < 180 indicated good performance, a 120 ≤ ACI < 160 indicated moderate effectiveness, and an ACI < 120 indicated ineffectiveness.

### Isolation and purification of *E*. *tenella *at different developmental stages

Healthy 14-day-old AA broilers were orally inoculated with 50,000 sporulated oocysts of *E*. *tenella*. From 7 to 9 dpi, oocysts were collected from the faecal samples using the saturated sodium chloride flotation method. Oocyst purification was achieved through gradient centrifugation with varying concentrations of sodium hypochlorite [18].

The isolation and purification of sporozoites were conducted in accordance with our previously published methods [19]. Briefly, the oocysts were disrupted using a tissue homogeniser (MagNA Lyser; Roche). Sporozoites were subsequently released through enzymatic digestion and finally collected using a G3 sintered glass funnel.

The methods for the isolation and purification of merozoites were adapted from those described by Wang et al. [20]. Briefly, 14-day-old healthy AA broilers were orally infected with 1 × 10^5^ sporulated *E*. *tenella* oocysts, and at 5 dpi, merozoites were isolated from caecal tissue through sequential homogenisation, red blood cell lysis (fivefold the pellet volume) and Percoll (17,089,101; Cytiva) gradient centrifugation.

The isolation and purification of gametes followed a method similar to that used for merozoites [21, 22]. At 144 h post-*E*. *tenella* infection, gametes were extracted from caecal mucosal tissue through tissue homogenisation, enzymatic digestion and Percoll density gradient centrifugation, followed by lysis using TRIzol (15,596,026; Invitrogen) for transcriptome sequencing (stored at − 80 °C).

### Preparation of *I. butyriciproducens* solutions

*I*. *butyriciproducens* was cultured anaerobically in Hungate tubes with anaerobic broth medium (CM1513; LAND BRIDGE) at 37 °C, passaged at a 1:100 dilution, and quantified at OD_600_ = 1 (1 × 10^9^ CFU/mL). Bacterial colonisation was achieved via cloacal enema, with 1 × 10^9^ CFU or 1 mL of culture supernatant (OD_600_ = 1) administered per chicken. For in vitro experiments, bacterial cells (BC) or culture supernatant (CS) were diluted to 50%, 20% and 10% in DMEM (11,320,033; Gibco) supplemented with 10% FBS (12003C; Sigma).

### Cell culture

DF-1 cells were cultured in DMEM (10% FBS, 1% penicillin‒streptomycin) in 25 T culture flasks (430,639; Corning). For other experiments, the cells were seeded onto a 48-well plate (351,178; Corning).

### qPCR

Total RNA was extracted from caecal tissue using TRIzol reagent (15,596,026; Invitrogen). Complementary DNA (cDNA) was synthesised using M-MLV reverse transcriptase (M1705; Promega) with random hexamer and oligo (dT)_15_ primers. qPCR amplification was performed using SYBR Green (D7262; Beyotime), and gene expression levels were normalised to those of *Et18S*. Relative gene expression in infected chickens compared with that in mock controls was calculated using the 2^−ΔΔCT^ method. The primers used are listed in Table 2. Sampling time points (3.5, 5.5 and 7 dpi) were selected on the basis of the methodology of Gaboriaud [13] to quantify *E*. *tenella* at distinct developmental stages.

**Table 2:** Primers for qPCR

Primer name	Forward_sequence (5' to 3')	Reverse_sequence (5' to 3')
*Et18S*	CTGATGCATGCAACGAGTTT	GACCAGCCCCACAAAGTAAG
*MZ80*	TTTCGCCGCATGATCATAT	CCACAACATACTCAGCACCTGC
*Etfoa1*	TCTCGCATTCCTCACAGATG	ATTTCGCCTTGTGGATGAAC
*Etgam56*	AGTGGCTGGAGAACTTCGTG	ATGCGGTTCGTGATCATGTC
*EtGFAT*	GGGCGATGGGCAGCTTACTACAAC	TCAAATGAACGCCCAAGGTCCAG
ETH_00000580	GGGAAGGAAGCGTTTGAGATGG	CCGAGGAGGCACCTGAGTTG
ETH_00001620	GTAGCAGCAACTCACACCACTC	GCAGCAGCAGCAGCAGAC
ETH_00002520	GCTCAAGATGTCCGTCACCTTC	TCCATTTCGTCCATACCCTCTCC
ETH_00003400	GCAACGGAGAGCAGCAGTG	CAGCAGCAGCAGCGTCATC
ETH_00008605	GCCTCGTCCTCCGCCAAG	GCTGCTGCTGCTGCTGTC
ETH_00008640	GCAGCAGCAGCAGACGATTC	CAGCAGCAGCAGCAAGAAGAG
ETH_00010290	AGCCCAGCAAGCCCATGTG	CCGCCAGCCTCCTTCTTCTC
ETH_00011565	TGCTTGCTGCTGATCTTTGGAG	GAGGTGCTGCTGCGTGAAG
ETH_00011955	TGCTGCTGCTGCTGCTGAG	TGCTGCTCGTCGTCGGTTC
ETH_00012295	GCAGCAGCAGCAGCAGTTG	TTCTCCTCCAGCGTCTCCATTC
ETH_00013240	CAGCAGCAGCAGCAGCAG	GCCGTTCGAGAGCGTTATCAG
ETH_00015050	AGCAGCAGCAGCAACAACAG	GCCCGCCGAGCAGTAACC
ETH_00016095	GCAGCAGCAGCAGCGTTAG	AGGCAGCAGCAGCACCAG
ETH_00017025	AGGTTGCTCATTGCGTCTTCATC	TTCCGCCTCTGCTCTTCCATC
ETH_00017345	CTGCTGCTGCTGCTGCTG	TGCTGCTGCTGCTTGTTGTC
ETH_00017570	AACAAGTTCCACGACGACAAGAG	TCCAGCAGGCTCGAAAGGTC
ETH_00019650	CCCACCTCCCTCTCAAAGACATC	TGCTGCTGCGGCTCACTAC
ETH_00020580	CGGCTGCTTCTTCTGCTTTCC	CGAGGGTGGCGTACAAGTTTC
ETH_00024980	TGCTTGGTGCTGCCGAGAG	CTGCTGCTGCTGCTGCTG
ETH_00025240	CGACACAGTTGATGCGAGGAG	GTGGATGCTGCTCTGGAGAATC
ETH_00026020	GCCAACTTCCTCGTCGTCAAC	CAAACACTCGCCGCAGGTTC
ETH_00026155	CGCTGTTGTTGATGTGGAATTTGAG	GCTGCTGCTGCTGCTCTTC
ETH_00028045	CAGAAGCAGCAGCAGCAGATG	ACAGCCGCAGCACAGAGC
ETH_00028885	GCAGCAGCAGTAACAGCGATAG	CTCTCCACCTCAACACCTCCTG
ETH_00029085	ACTTGAGAACTCGGAAGCATTGTC	TACTTGCGGCGGAACACCTC
ETH_00030540	GCTGCTGCTGCTGCTTCTG	CTGCTGCTGCTTCTGCTTCTG
ETH_00033310	TTCAAGTGCGGCATCAACTACC	GCGATGGCGGTGCTGTTG
ETH_00033590	TCGGAAGAAGAATGGGAGAAGACAG	AGGAAGCACAGCAGCAGAGAC
ETH_00039710	AGAATAGATGAGTTGCTGCGGAAG	GTGCTCGTGCTGCTGCTG
ETH_00042545	AGCAGCCGCAGTCGAGAAG	CAGTCGTGGTCGTAGGGAAGTC

### Sporozoite invasion assay

The sporozoite invasion assay was conducted according to the method described by S. Hessenberger [23], using CFSE-positive cell proportions to quantify *E*. *tenella* sporozoite invasion rates. DF-1 cells were seeded at 1 × 10^5^ cells/well in a 48-well plate and cultured in growth medium (DMEM supplemented with 10% FBS and 1% penicillin‒streptomycin) until they reached 80% confluency. To assess the direct effect of the antibiotic cocktail (ABX) on sporozoite invasion, the growth medium was then removed, and the cells were gently washed with PBS. The cells were subsequently incubated in fresh, antibiotic-free DMEM (10% FBS) supplemented with serially diluted ABX at the following concentrations: A (100% of the in vivo gavage concentration), B (10%), C (1%) and D (0.1%). The control group (CON) received antibiotic-free DMEM (10% FBS) only. Purified *E*. *tenella* sporozoites were labelled with 5 μM CFSE (37 °C, 20 min). Thereafter, 5 × 10^4^ labelled sporozoites were added to each well. The cocultures were incubated (37 °C, 5% CO₂, 12 h), washed with PBS to remove noninvaded sporozoites, detached with 0.25% trypsin and analysed via flow cytometry to determine the proportion of CFSE-positive cells.

### Evaluation of merozoite mortality

Purified *E*. *tenella* merozoites were quantified using a haemocytometer, and 100,000 merozoites per well were seeded in a 48-well plate. Merozoites were incubated in DMEM supplemented with 10% FBS, 1% penicillin‒streptomycin and various antibiotic concentrations at 37 °C with 5% CO_2_ for 12 h. After incubation, the merozoites were collected into 5 mL polystyrene tubes, centrifuged at 1500 rpm for 10 min, and resuspended in 100 μL of 1 × binding buffer. The samples were stained with 7-AAD (559,925; BD), vortexed and incubated at room temperature in the dark for 15 min. After 400 μL of 1 × binding buffer was added, merozoite mortality was analysed via flow cytometry on the basis of 7-AAD uptake, which indicated loss of membrane integrity.

### Lymphocyte isolation and flow cytometry

Lymphocytes were isolated from the blood, caecal tonsils and spleen. Peripheral blood lymphocytes were isolated from 1 mL of fresh blood per chicken using a commercial kit (P8740; Solarbio, China). Caecal tonsils and spleen tissues were homogenised and filtered through a 200-mesh cell strainer, and lymphocytes were isolated using a Solarbio kit (P9120; Solarbio, China). The cell count was adjusted to 1 × 10^6^ cells per 100 µL.

For T cell analysis, 1 µL each of CD3-FITC, CD4-APC and CD8-PE antibodies (8200–02, 8210–11 and 8220–09; SouthernBiotech) was added sequentially to 100 µL of cell suspension, incubated at 4 °C in the dark for 30 min, and then washed twice with PBS. For B cell analysis, 1 µL of a PE-conjugated anti-Bu-1 antibody (8395–09; SouthernBiotech) was added, incubated under the same conditions and then washed twice with PBS.

For negative selection, spleen lymphocytes from healthy chickens were labelled with APC-conjugated anti-CD4 and PE-conjugated anti-CD8 antibodies. After sorting, 1 × 10^6^ CD4^−^ and 1 × 10^6^ CD8^−^ cell complexes were obtained for coculture with *I*. *butyriciproducens*.

### Enzyme‑linked immunosorbent assay

The levels of chicken serum IFN-γ and IL-17, as well as IFN-γ levels in the supernatant from the negative selection assay, were measured using enzyme-linked immunosorbent assay (ELISA) kits (LV90230, LV90252; Animal Union, China) according to the manufacturer’s instructions.

### Histopathological analysis

Caecal tissues were fixed in 4% paraformaldehyde for > 48 h, followed by sequential dehydration in 70–100% ethanol, xylene treatment, paraffin embedding and sectioning at a thickness of 3 µm. Sections were deparaffinized, hydrated and stained with haematoxylin and eosin (HE staining). Caecal lesion scoring was performed in a double-blind manner by coccidia research experts.

To perform the fluorescence in situ hybridisation (FISH) experiments, advanced π-FISH technology developed by Huazhong Agricultural University was employed. Probe pairs targeting *EtGFAT* were designed by Spatial FISH, Co., Ltd. The hybridisation process, similar to that of traditional FISH, included dehydration, digestion, fixation, hybridisation at 40 °C and elution. For detailed protocols, refer to Tao et al. [24].

### Transmission electron microscopy

Caecal tissue was trimmed to 1 mm^3^ and fixed sequentially in 2.5% glutaraldehyde and 1% osmium tetroxide, each for 3 h, followed by rinsing in 0.1 M phosphate buffer. The tissues were dehydrated in graded ethanol and acetone series, incubated with a 1:1 EPON812-acetone mixture, embedded in EPON812 resin and polymerised at 60 °C for 48 h. Ultrathin Sects. (70 nm) were cut using a Leica UC7 microtome, stained with 2% uranyl acetate and lead citrate for 15 min each, air-dried overnight and examined using a transmission electron microscope (FEI, TECNAI G2 20 TWIN).

### DNA extraction, PCR amplification and sequencing

To characterise the microbial community structure, caecal content samples were collected for 16S rRNA gene sequencing at two time points: At 0 dpi (immediately prior to *E*.* tenella* challenge), samples were collected from the CON and ABX groups to validate the establishment of the antibiotic-induced dysbiosis model. Six biological replicates (*n* = 6) were included per group. At 6 dpi (during the peak gametogony stage), samples were collected from the CON, ABX and FMT groups with the specific aim of identifying microbial taxa associated with altered parasitic development. Ten biological replicates (*n* = 10) were included per group.

All the samples were collected individually, immediately snap-frozen in liquid nitrogen, and stored at − 80 °C until DNA extraction. Chicken caecal contents were collected and stored at − 80 °C until processing. Total genomic DNA was extracted using the SDS method, and its concentration and purity were assessed on 1% agarose gels. The V3–V4 region of bacterial 16S rDNA was amplified using barcoded PCR primers (forward: ACTCCTACGGGAGGCAGCA; reverse: GGACTACHVGGGTWTCTAAT). The sequencing libraries were prepared using the NEBNext® Ultra™ IIDNA Library Prep Kit (Cat No. E7645) following the manufacturer’s instructions. Library quality was evaluated using a Qubit® 2.0 fluorometer (Thermo Scientific) and an Agilent Bioanalyzer 2100 system. Sequencing was performed on an Illumina NovaSeq platform, generating 250 bp paired-end reads. Quality control and sequencing were conducted by Novogene Bioinformatics Technology Co., Ltd.

### RNA extraction, library construction and sequencing

To obtain sufficient quantities of gametes for transcriptome analysis, a total of 90 chickens were used (30 per group: CON, ABX and FMT). At 6 dpi, gametes were isolated from the caecal mucosal tissue of each group. Gametes from every 10 chickens within the same treatment group were pooled to form one biological.

replicate, resulting in three independent biological replicates per group and a total of nine samples for RNA-seq. The purified gametes were immediately lysed in TRIzol reagent and stored at − 80 °C until RNA extraction.

Total RNA was purified using poly-T oligo magnetic beads, and mRNA was fragmented with divalent cations at elevated temperatures in first-strand synthesis reaction buffer (5 ×). First-strand cDNA was synthesised with random hexamer primers and M-MLV reverse transcriptase, followed by second-strand synthesis using DNA polymerase I and RNase H. Overhangs were blunted, and adaptors with hairpin loops were ligated. cDNA fragments of 370–420 bp were size-selected using the AMPure XP system (Beckman Coulter). PCR amplification was performed with Phusion High-Fidelity DNA polymerase and primers, and the products were subsequently purified with AMPure XP. Library quality was assessed on an Agilent Bioanalyzer 2100. Indexed samples were clustered on a cBot using the TruSeq PE Cluster Kit v3-cBot-HS (Illumina) and sequenced on an Illumina NovaSeq platform, generating 150 bp paired-end reads (Novogene Bioinformatics Technology Co., Ltd).

### Bioinformatic analysis of RNA-seq data

Raw read counts were processed and analysed using the DESeq2 package (version 1.20.0) in R. Genes with an adjusted p value (*padj*) < 0.05 and absolute log2-fold change |log2FC|> 1.0 were consideredto be significantly differentially expressed.

### Metabolomic analysis of *I. butyriciproducens* culture supernatant

*I*. *butyriciproducens* was cultured in anaerobic broth medium at 37 °C for 48 h until the desired density was achieved. The culture was subsequently centrifuged to separate the bacterial cells from the supernatant, which was subsequently filtered to obtain a sterile, cell-free liquid. The filtered supernatant was aliquoted into 10 mL centrifuge tubes and sent to Novogene Bioinformatics Technology Co., Ltd., for nontargeted metabolomics analysis using liquid chromatography‒mass spectrometry (LC‒MS). The resulting data were processed and analysed for metabolite identification and quantification.

### Determination and analysis of short-chain fatty acid content

Short-chain fatty acid (SCFA) content in chicken caecal contents was determined using a mixed standard stock solution of six SCFAs (acetic acid, propionic acid, isobutyric acid, butyric acid, isovaleric acid and valeric acid) and caproic acid, each at 100 mg/mL, prepared in water or ether. A calibration curve was generated by diluting the stock solutions to concentrations ranging from 0.02 to 500 µg/mL, with 4-methylvaleric acid (375 µg/mL) as the internal standard (IS). The samples were homogenised with water and glass beads, centrifuged and extracted with phosphoric acid, IS and ether. After centrifugation, the supernatants were analysed via gas chromatography‒mass spectrometry (GC‒MS) using a Thermo Fisher Trace 1310 GC and ISQ LT MS. The SCFA content was calculated using specific formulas, and the reagents were sourced from Sigma‒Aldrich, Sinopharm, and Titan.

### Bioinformatics analysis of the sequencing data

The raw sequencing reads were processed using fastp (version 0.14.1; Link 1) with the parameters -W4–M20 for quality filtering and adapter removal. The primer sequences were trimmed using cutadapt (Link 2) to generate high-quality paired-end clean data. The paired-end reads were merged using the usearch-fastq_mergepairs tool (version 10; Link 3). Denoising of the merged sequences was performed using the DADA2 (or deblur) pipeline to generate amplicon sequence variants (ASVs). Taxonomic annotation of representative ASV sequences was conducted using the SILVA database. Alpha diversity indices for commensal bacterial communities were calculated on the basis of ASV abundance profiles using the search-alpha_div tool (version 10; Link 3). All statistical analyses and visualisations—including bar plots, heatmaps and ternary diagrams based on relative taxonomic abundances—were generated using R software.

### Statistical analysis

To ensure the robustness of the data, biological replicates (independent samples) and technical replicates (repeated measurements of the same sample) were included in all the experiments. Each data point represents an independent sample. Statistical analysis was performed on the basis of these replicates to account for biological and technical variability.

All the statistical analyses were conducted using GraphPad Prism 9 (GraphPad Software, San Diego, CA, USA). The data are expressed as the mean ± standard deviation (SD). Normality was assessed by the Shapiro‒Wilk test. For normally distributed data, one-way ANOVA was used for multigroup comparisons, with Tukey’s post hoc test for pairwise comparisons as appropriate. For two-group comparisons, an unpaired two-tailed Student’s *t* test was applied. When the data were nonnormally distributed, the Kruskal‒Wallis test with Dunn’s multiple comparisons was used for multigroup analyses, and the Mann‒Whitney *U* test was used for two-group comparisons. *p* values are denoted as follows: *p < 0.05; **p < 0.01; ***p < 0.001; and ****p < 0.0001.

## Results

To elucidate the role of the caecal microbiota in *E*. *tenella* development, we used ABX to establish an infection model under dysbiotic conditions. To our knowledge, this study represents the first investigation into the influence of the microbiota on *E*. *tenella* development and infection in an ABX-induced pseudogerm-free chicken model. To validate the feasibility of this model, we evaluated the effect of ABX on *E*. *tenella* sporozoite invasion. Briefly, CFSE-labelled sporozoites were coincubated with DF-1 cells for 12 h in the presence of varying ABX concentrations, and flow cytometry was used to quantify the proportion of CFSE-positive cells (Fig. 1a, b). The results demonstrated that only the highest ABX concentration, which was equivalent to the dose used in the animal experiments, directly impaired cell physiology and significantly inhibited sporozoite invasion (*p* < 0.05; Fig. 1c). However, this inhibitory effect observed with the highest concentration is pharmacologically irrelevant in vivo because of its rapid metabolism and degradation and therefore does not provide meaningful insight for evaluating the anti-infection efficacy of ABX. In contrast, lower concentrations (10%, 1% and 0.1% of the experimental dose) had no detectable effect on the invasion process.

To further examine the impact of ABX on *E*. *tenella* merozoites, we incubated isolated and purified merozoites with the same gradient of ABX concentrations (gavage concentration, as well as 10%, 1% and 0.1% of this concentration) for 12 h. After incubation, the proportions of necrotic merozoites were evaluated using 7-AAD staining (Fig. 1d). In comparison with the control, ABX had no significant effect on merozoite viability at any concentration tested. On the basis of these findings, we conclude that ABX does not directly affect either sporozoites or merozoites, thereby validating its suitability for establishing a dysbiotic *E*. *tenella* infection model.

One-day-old AA broilers were treated with ABX for 14 days, followed by infection with 50,000 sporulated *E*. *tenella* oocysts per chick (ABX group; Fig. 1e). At 0 days post-infection (dpi), the caecal microbiota structure of the ABX and CON groups was analysed using amplicon sequencing. First, the number and composition of amplicon sequence variants (ASVs) were compared between the ABX and CON groups. The bar chart (Fig. 1f) illustrates the ASV distribution across taxonomic levels for each sample, revealing a significantly higher ASV count in the CON group than in the ABX group, indicating that ABX treatment markedly reduced microbiota diversity. Across all the taxonomic levels, the CON group exhibited greater ASV richness, particularly at lower ranks such as family, genus and species, whereas the ABX group showed a pronounced reduction. A Venn diagram further revealed that some of the ASVs overlapped between the two groups: the CON group uniquely contained 3431 ASVs (81.79%), the ABX group contained 675 unique ASVs (16.09%) and only 89 ASVs (2.12%) were shared between them. These results demonstrate that ABX treatment significantly altered the microbiota composition, resulting in substantial structural differences and a notable reduction in diversity in the ABX group compared with those in the CON group.

To comprehensively assess alpha diversity, we employed the Chao1 and observed species indices to quantify species richness, the Shannon and Simpson indices to measure species diversity, Faith’s phylogenetic diversity (PD) index to evaluate phylogenetic diversity, Pielou’s evenness index to determine species evenness and Good’s coverage index to estimate species coverage (Fig. 1g). Data analysis was conducted using QIIME2 software, with statistical significance determined by the Kruskal‒Wallis test. The results revealed significant differences between the ABX and CON groups across all indices—Chao1, Simpson, Shannon, Pielou’s evenness, observed species, Faith’s PD and Good’s coverage. These findings indicate that compared with no treatment, antibiotic treatment induced profound alterations in the richness, diversity and phylogenetic structure of the caecal microbiota.

To supplement the alpha diversity assessment and further validate the compositional disparities between the experimental groups, beta diversity analysis of the caecal microbiota was performed to compare the ABX and CON groups. Antibiotic intervention induced significant restructuring of the caecal microbiota, culminating in a distinct community structure relative to that of the untreated CON group. Both principal coordinate analysis (PCoA) and nonmetric multidimensional scaling (NMDS) demonstrated pronounced segregation between groups in terms of microbial community structure. The PCoA ordination revealed that principal coordinates 1 and 2 accounted for 74.5% and 13.6% of the total variance, respectively (Fig. 1h), while the NMDS analysis yielded a stress value of 0.0457, indicating high model fidelity (Fig. 1i). These beta diversity metrics corroborate the alpha diversity findings, collectively demonstrating that compared with undisturbed microbial communities, antibiotic-induced dysbiosis substantially diminished both taxonomic richness and ecological complexity within the caecal microbiota.

**Fig. 2:**
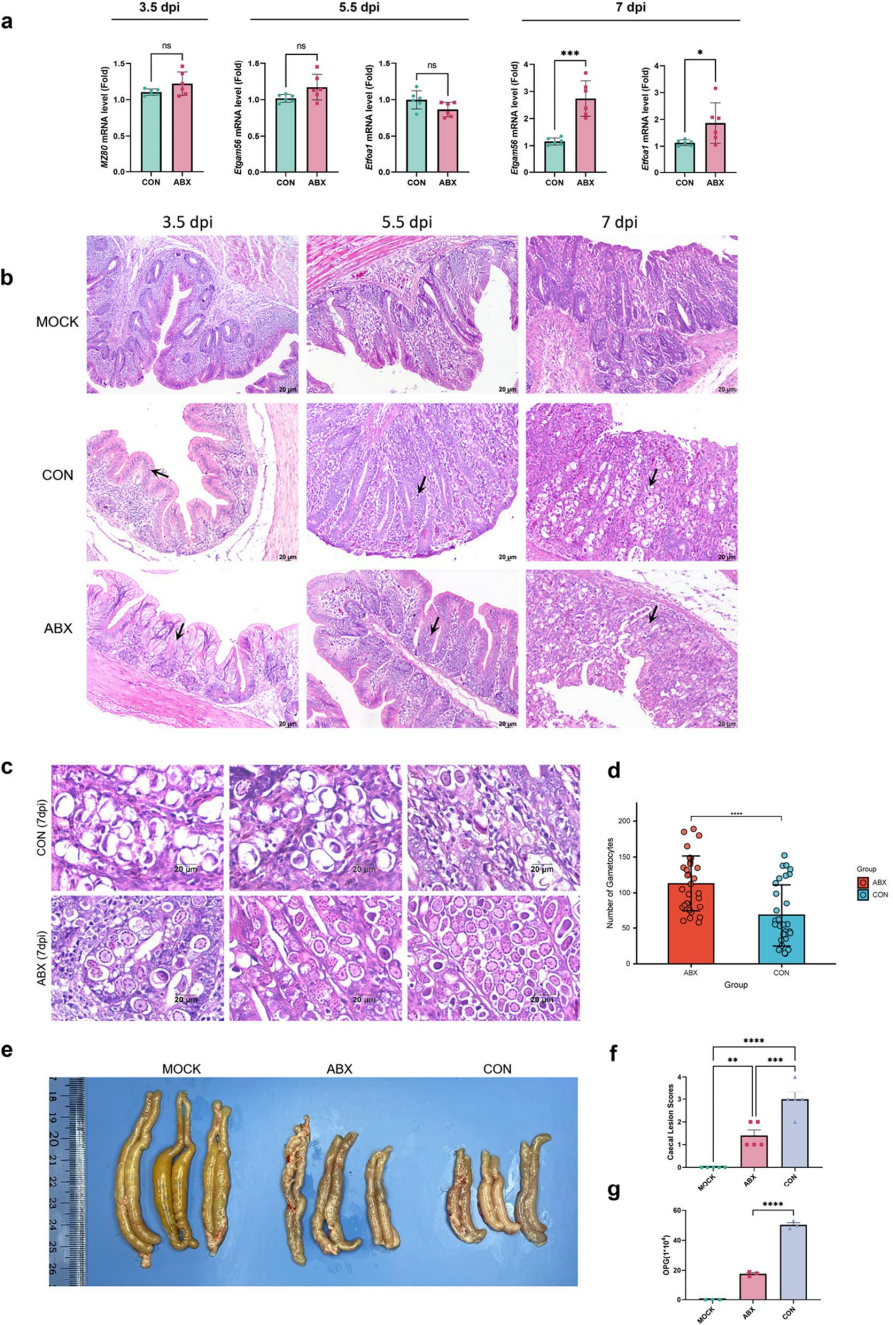
Effects of caecal microbiota dysbiosis on *E*. *tenella* development and caecal pathology. **a** Relative expression levels of stage-specific *E*. *tenella* genes—*MZ80* (merozoite-specific), *Etgam56* (macrogamete-specific) and *Etfoa1* (microgamete-specific)—were normalised to those in the CON group. The expression of the macrogamete-specific gene *Etgam56* significantly increased at 7 dpi (***p < 0.001), whereas the expression of the microgamete-specific gene *Etfoa1* also increased at 7 dpi (*p < 0.05) in the ABX group compared with the CON group. **b** HE staining of caecal tissues from the MOCK, CON, and ABX groups at different infection stages (3.5, 5.5 and 7 dpi). The arrows indicate the corresponding *E. tenella* stages observed in both the CON and ABX groups at each time point. Representative images are shown for each group. **c** H&E staining of caecal tissues at 7 dpi, comparing the ABX and CON groups. The number of parasitic gametes was greater in the ABX group. **d** Quantification of parasites in different developmental stages in the caeca of the ABX and CON groups at 7 dpi. Each data point represents the parasite count from nonoverlapping fields. Compared with the CON group, the ABX group presented a significantly greater number of gametes (****p < 0.0001). **e** Representative macroscopic images of chicken ceca from the MOCK, ABX and CON groups, illustrating apparent differences in lesion severity. **f** The lesion scores of the ceca from the MOCK, CON and ABX groups were significantly greater in the ABX group than in the MOCK and CON groups (***p < 0.001). **g** Quantification of OPG in the faecal samples from the CON and ABX groups at 7 dpi. Each group consisted of pooled faecal samples from 5 chickens, with counts obtained from five randomly selected sampling points per sample, indicating a reduction in oocyst shedding in the ABX group (***p < 0.001)

To investigate the impact of caecal microbiota dysbiosis on *E*. t*enella* development across various life stages, we performed quantitative PCR (qPCR) on caecal tissue samples to assess the relative expression of stage-specific genes. These genes included the merozoite-specific gene *MZ80* (ETH_00011530), the macrogamete-specific gene *Etgam56* (ETH_00007320) and the microgamete-specific gene *Etfoa1* (ETH_00025255) (Fig. 2a). We used *Et18S* as an internal reference gene, normalised gene expression to that in the CON group and applied the 2^−ΔΔCT^ method, where ΔCT represents the difference between the CT value of the target gene and the average CT value of *Et18S*. Comparative analysis of gene expression levels revealed no significant differences in *MZ80* expression between the ABX and CON groups at 3.5 dpi. Similarly, at 5.5 dpi, the expression levels of the gametocyte-specific genes *Etgam56* and *Etfoa1* in the ABX group did not significantly differ from those in the CON group. However, at 7 dpi, compared with the CON group, the ABX group presented significantly elevated expression levels of *Etgam56* (*p* < 0.01) and *Etfoa1* (*p* < 0.05), suggesting that caecal microbiota dysbiosis may disrupt gene expression during the gametogony stage.

Additionally, HE staining was employed to assess parasite localisation and caecal pathology at various stages postdysbiosis (Fig. 2b). At 3.5 dpi, the parasite distribution was comparable between the ABX and CON groups, with minimal parasite presence in the intestinal epithelial cells; however, the CON group exhibited more pronounced inflammatory cell infiltration and deeper caecal folds. At 5.5 dpi, the ABX group displayed fewer parasites in the caecum and preserved caecal fold architecture, whereas the CON group exhibited haemorrhagic foci and more severe lesions. At 7 dpi, the CON group demonstrated extensive oocyst accumulation and haemorrhagic areas, whereas the ABX group primarily showed gametocyte accumulation with relatively milder lesions (Fig. 2c).

To further validate the differences in gametocyte abundance at 7 dpi, we quantified parasites in ten randomly selected fields from three HE-stained samples per group (Fig. 2d). The ABX group tissues exhibited a substantial number of gametocytes during the oocyst-shedding phase, whereas the CON group tissues were characterised predominantly by oocysts. Quantitative analysis revealed a statistically significant difference between the two groups (*p* < 0.0001), suggesting that caecal microbiota dysbiosis may influence *E*. *tenella* gametogony.

In terms of overall caecal lesion severity (Fig. 2e), the MOCK group tissues exhibited normal caecal morphology with intact luminal contents, whereas the CON group tissues had shortened ceca, thickened intestinal walls and white caseous necrotic debris. The ABX group demonstrated the least severe lesions (Fig. 2f). Analysis of OPG counts at 7 dpi (Fig. 2g) revealed a significant reduction in oocyst shedding in the ABX group (*p* < 0.0001), further supporting the hypothesis that caecal microbiota dysbiosis increased the number of gametocytes and attenuated oocyst production.

**Fig. 3:**
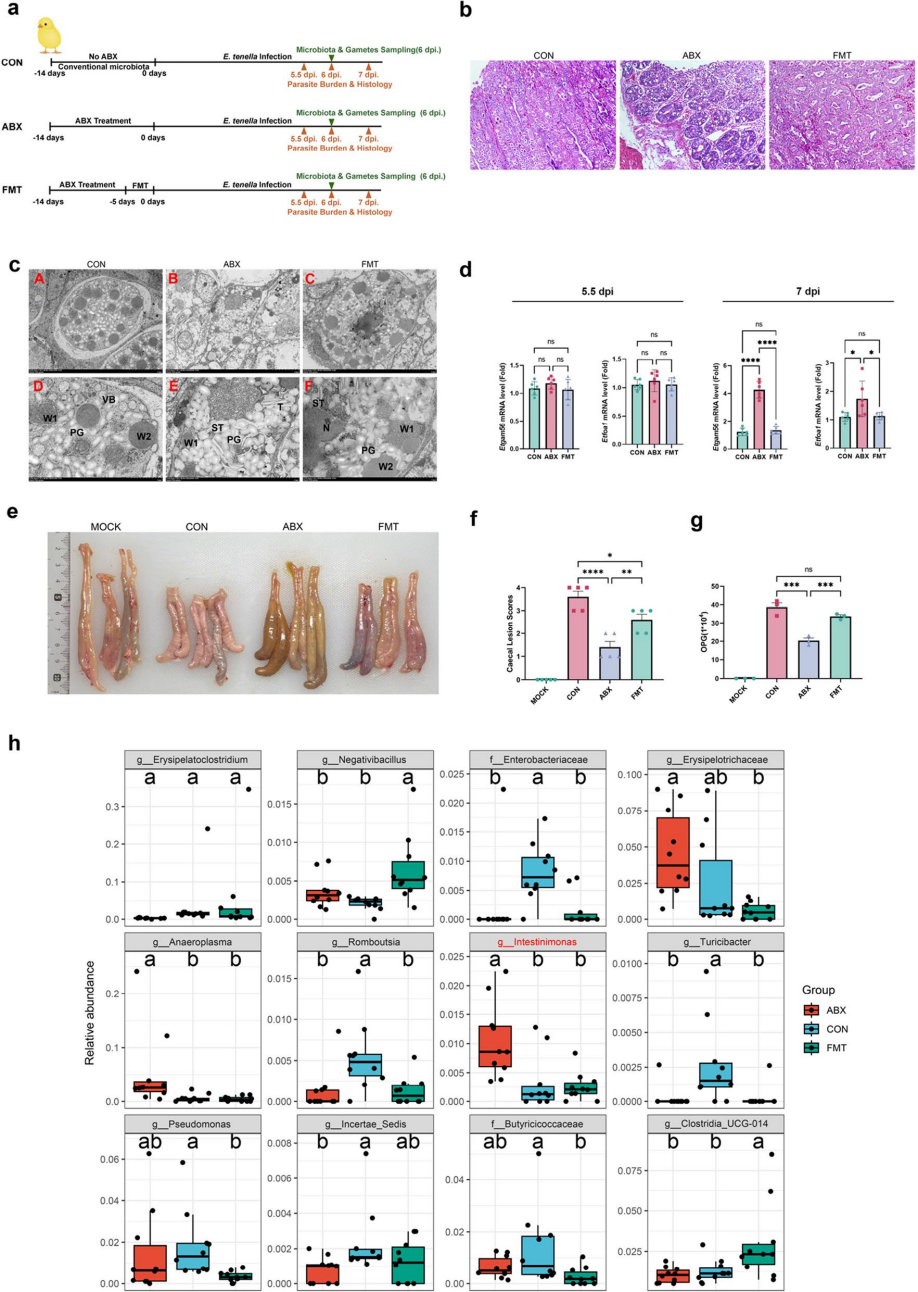
FMT abrogates *E. tenella* developmental arrest, and *Intestinimonas* is a pivotal genus mediating this effect. **a** AA broiler cockerels received ABX treatment for 14 days to induce dysbiosis, followed by either FMT or continued ABX treatment, with infection by *E*.* tenella* at 0 dpi. **b** H&E staining of caecal tissues from the CON, ABX and FMT groups at 7 dpi. Dysplastic abnormalities and an increase in parasitic stages were evident in the ABX group, whereas FMT partially restored the caecal community structure. Representative images are shown. **c** Transmission electron microscopy (TEM) images of gametocyte and oocyst structures in caecal tissue at 7 dpi for the CON, ABX and FMT groups. The observed structures include wall-forming bodies (W1, W2), nuclei (N), vacuolated bodies (VB), polar granules (PG) and stieda bodies (ST). **d** Relative expression levels of stage-specific *E*. *tenella* genes (*MZ80*, *Etgam56* and *Etfoa1*) at 5.5 dpi and 7 dpi. Significant differences in gene expression were observed at 7 dpi, with *Etgam56* and *Etfoa1* elevated in the ABX group compared with the CON group (*p < 0.05, **p < 0.01, ***p < 0.001). **e** Representative macroscopic images of chicken ceca from the MOCK, CON, ABX and FMT groups. **f** Caecal lesion scores of the MOCK, CON, ABX and FMT groups at 7 dpi. Compared with the MOCK and CON groups, the ABX group had significantly lower lesion scores. **g** Quantification of OPG in faecal samples at 7 dpi, comparing the CON, ABX, and FMT groups (***p < 0.001). **h** Comparison of the relative abundance of selected microbial genera and families in the caecal contents of the CON, ABX and FMT groups at 6 dpi. Differentially abundant taxa were identified using random forest cross-validation (RFCV). The letters above the bars indicate significant differences between groups

To investigate the impact of caecal microbiota dysbiosis on the development of *E*. *tenella*, we performed a microbiota reconstitution experiment (Fig. 3a). At 14 days of age (0 dpi), the caecal contents from the CON, ABX and FMT groups (*n* = 6 per group) were subjected to amplicon sequencing (Supplementary Fig. 1). Alpha diversity analysis demonstrated that the Chao1, Simpson, Shannon, observed species, Faith’s phylogenetic diversity (PD) and Good’s coverage indices were significantly greater in the FMT group than in the ABX group and did not differ significantly from those in the CON group. NMDS analysis further revealed that the microbial communities in the CON and FMT groups clustered closely, exhibited minimal variation and were distinctly separated from those in the ABX group. These findings indicate successful reconstitution of the caecal microbiota in FMT-treated AA broilers.

To assess gametocyte development, caecal tissue samples collected at 6 dpi were subjected to HE staining. The results demonstrated that the CON and FMT groups harboured abundant gametocytes within the intestinal glands, whereas the ABX group exhibited a predominance of severely shrunken and malformed structures that were often unidentifiable as discrete gametocytes, indicating a catastrophic failure in morphological development (Fig. 3b). To further investigate these morphological differences, transmission electron microscopy (TEM) was employed (Fig. 3c). TEM analysis revealed that gametocytes in the ABX group were severely deformed and characterised by irregularly distributed wall-forming bodies, indistinct nuclei, dispersed perinuclear radial structures and incomplete polysaccharide granules. In contrast, gametocytes in the CON and FMT groups displayed normal morphology, featuring intact cellular structures, clearly defined nuclei, well-organised wall-forming bodies, complete polysaccharide granules and distinct perinuclear radial structures.

Additionally, we evaluated the expression levels of the gametocyte-specific genes *Etgam56* and *Etfoa1* in *E*. *tenella* (Fig. 3d). At 5.5 dpi, the expression levels of *Etgam56* and *Etfoa1* in the FMT group were comparable to those in the CON group, whereas the ABX group presented significantly higher expression levels. However, at 7 dpi, *Etgam56* expression in the ABX group was significantly greater than that in both the CON group (*p* < 0.01) and the FMT group (*p* < 0.001), while *Etfoa1* expression was also significantly greater in the ABX group than in the CON and FMT groups (*p* < 0.05). No significant differences were observed between the FMT and CON groups, demonstrating that FMT restored gene expression to levels consistent with those observed in conventional infection conditions. Crucially, this sustained and significantly elevated expression of gametocyte-specific genes in the ABX group at 7 dpi does not indicate restored development but rather suggests a failure to successfully complete the gametogony process and transition to oocyst formation. This transcriptional dysregulation aligns with the observed accumulation of malformed gametocytes (Fig. 3b, c) and the subsequent reduction in oocyst shedding (Fig. 3g), providing a molecular explanation for the parasitic developmental arrest induced by caecal microbiota dysbiosis.

Macroscopic examination of the caecal tissue (Fig. 3e) revealed thickened caecal walls, shortened lengths and extensive white necrotic lesions, characteristic of coccidiosis, in the CON group. Compared with the CON group, the FMT group displayed similar lesion severity, while the ABX group exhibited the mildest lesions. Lesion severity scoring (Fig. 3f**)** revealed significantly higher scores in the FMT group than in the ABX group (*p* < 0.01). OPG counts at 7 dpi (Fig. 3g) revealed that the ABX group had significantly lower OPG levels than the CON group did (*p* < 0.001). In contrast, OPG levels were significantly higher in the FMT group than in the ABX group (*p* < 0.001) and were not significantly different from those in the CON group.

Finally, we employed a random forest regression algorithm to analyse the relative abundance and compositional differences in the caecal microbiota among the CON, ABX and FMT groups at the gametogony stage (6 dpi). The 12 bacterial genera depicted represent the initial screening results and are the most representative differential taxa for further analysis (Fig. 3h). We subsequently refined the screening criteria to identify bacterial genera that were significantly more abundant in the ABX group but were not significantly different between the CON and FMT groups. *Intestinimonas* fulfilled these criteria, suggesting its potential role as a key inhibitor of *E*. *tenella* gametocyte development.

**Fig. 4:**
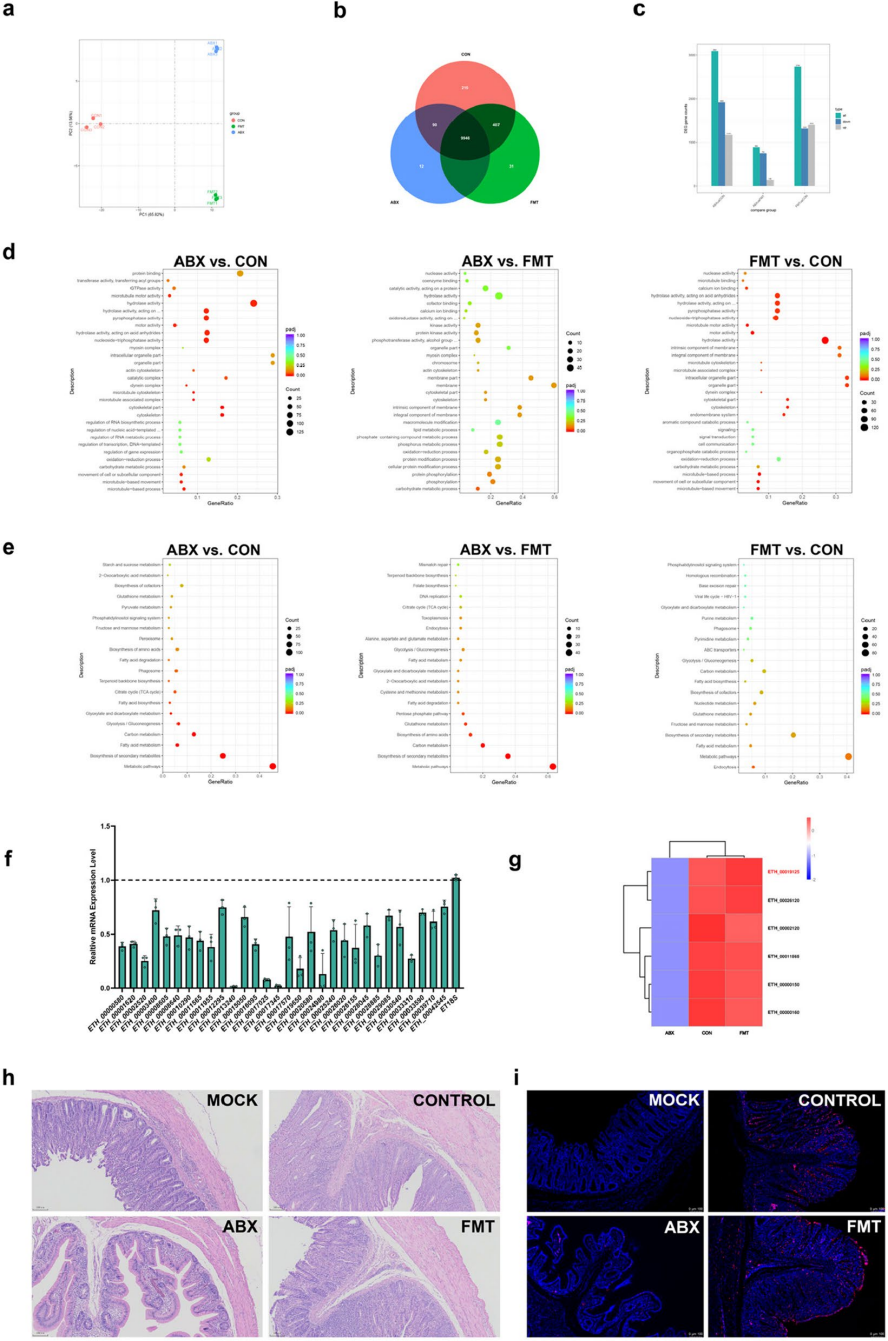
RNA-seq analysis of *E. tenella* gametes revealed that microbiota integrity regulates gamete gene expression, and *EtGFAT* is a key regulatory gene in this process. **a** The PCA plot illustrates variance in gene expression profiles among the control (CON), antibiotic-induced dysbiosis (ABX) and faecal microbiota transplantation (FMT) groups, highlighting distinct clustering patterns and significant transcriptomic differences between groups. **b** Venn diagram of RNA-seq data from the CON, ABX and FMT groups, illustrating shared and unique gene expression profiles among the control (CON), antibiotic-induced dysbiosis (ABX) and faecal microbiota transplantation (FMT) groups. A total of 9946 genes were coexpressed across all three groups, with 210 genes unique to CON, 12 unique to ABX, and 31 unique to FMT. **c** Differentially expressed gene (DEG) analysis among the CON, ABX, and FMT groups. DEGs were defined by an adjusted *p* value (*padj*) ≤ 0.05 and an absolute log2(fold change) ≥ 1.0. **d** Scatterplot of the results of the GO enrichment analysis of DEGs between groups. The bar chart shows the top 30 most significant GO terms. **e** Scatterplot of KEGG enrichment analysis results for DEGs across groups, represented by bar charts and bubble plots, with a significance threshold of *padj* < 0.05. **f** Thirty genes downregulated in the ABX group were randomly selected for qPCR validation. The qPCR results align with the RNA-seq findings, confirming the reliability of the transcriptomic data. **g** Clustering heatmap of six differentially expressed genes. Among the genes whose expression was downregulated in the ABX group compared with the CON group, 152 genes were annotated, while 95 genes whose expression was downregulated in the ABX group compared with the FMT group were annotated. The comparison of these sets revealed 23 genes whose expression was lower in the ABX group than in both the CON and FMT groups. Clustering analysis of these 23 genes further revealed six genes whose expression levels were similar between the CON and FMT groups. Notably, ETH_00019125 (*EtGFAT*) is involved in multiple biological processes, including “alanine, aspartate and glutamate metabolism”, “nucleotide sugar biosynthesis” and “amino sugar and nucleotide sugar metabolism”. **h** Paraffin embedding and pathological sectioning of caecal tissues at 6 dpi were conducted for each group, followed by H&E staining. **i** π-FISH Rainbow fluorescence in situ hybridisation was used to localise the *EtGFAT* gene in each group. Red fluorescence indicates *EtGFAT* gene expression, while blue fluorescence indicates DAPI-stained nuclei. The fluorescence intensity and distribution density reflect the expression level and localisation of the *EtGFAT* gene. Strong and densely distributed *EtGFAT* signals were detected in the CON and FMT groups, whereas weaker and more sparse fluorescence signals were detected in the ABX group

Previous experiments have demonstrated that caecal microbiota dysbiosis impairs macrogamete development in *E*. *tenella*. To identify the key genes involved, we isolated *E*. *tenella* gametes using Percoll gradient centrifugation. Three samples were collected from each group (CON, ABX and FMT), with gametes pooled from every 10 chickens to form one sample, resulting in a total of nine samples for transcriptome sequencing. Principal component analysis (PCA) of gene expression values (FPKM) revealed distinct clustering within each group and clear separation between groups (Fig. 4a). Venn diagrams further highlighted the genes that were shared and unique across the CON, ABX and FMT groups (Fig. 4b).

Differential gene expression analysis was performed for three pairwise comparisons: ABX vs. CON, ABX vs. FMT and FMT vs. CON. In the ABX vs. CON comparison, we identified 3091 differentially expressed genes (DEGs), comprising 1171 upregulated genes and 1920 downregulated genes. For the FMT vs. CON comparison, 2738 DEGs were detected, including 1413 upregulated genes and 1325 downregulated genes. In the ABX vs. FMT comparison, 890 DEGs were identified, with 752 upregulated and 138 downregulated genes (Fig. 4c).

Gene Ontology (GO) enrichment analysis was conducted on the DEGs (SupplementaryFig. 2), with the top 30 most significant terms visualised as bubble plots (Fig. 4d). In the ABX vs. CON comparison, the top enriched GO terms included “cytoskeletal part”, “cytoskeleton” and “microtubule-associated complex”. In the ABX vs. FMT comparison, the top enriched terms were “protein phosphorylation”, “phosphorylation” and “carbohydrate metabolic process”. In the FMT vs. CON comparison, the top enriched terms were “microtubule-based process”, “movement of cell or subcellular component” and “microtubule-based movement”.

Using the *Toxoplasma gondii* genome as a reference, we performed Kyoto Encyclopedia of Genes and Genomes (KEGG) pathway enrichment analysis on the DEGs (Fig. 4e). In the ABX vs. CON comparison, the most significantly enriched pathways included “metabolic pathways”, “biosynthesis of secondary metabolites” and “fatty acid metabolism”. Similarly, the ABX vs. FMT comparison highlighted pathways such as “metabolic pathways” and “biosynthesis of secondary metabolites”. In contrast, the FMT vs. CON comparison primarily involved “fatty acid metabolism”. These findings suggest that an intact caecal microbiota is essential for normal gamete gene expression.

To validate the RNA-seq results for *E*. *tenella* gametes, we randomly selected 30 downregulated genes from the ABX vs. CON comparison for qPCR validation. The qPCR results were consistent with the RNA-seq findings (Fig. 4f), thereby enhancing the reliability of the data. We hypothesised that specific genes involved in *E*. *tenella* gamete development are regulated by the caecal microbiota. To identify these genes, we focused on those whose expression was downregulated in the ABX vs. CON comparison and upregulated in the FMT vs. ABX comparison. Following the exclusion of genes with incomplete annotations, we employed clustering analysis to visualise the expression patterns of the overlapping genes (Fig. 4g).

We subsequently identified the *EtGFAT* gene (ETH_00019125; *Eimeria tenella* glucosamine: fructose-6-phosphate aminotransferase) as a candidate gene (SupplementaryFig. 3). Using fluorescence in situ hybridisation (FISH), we localised *EtGFAT* expression, which corresponded with the HE staining results (Fig. 4h). Notably, *EtGFAT* was highly expressed during the gametogony stage. Furthermore, its expression level varied with the status of the caecal microbiota, with expression significantly lower in the ABX group and restored to higher levels in the FMT group (Fig. 4i).

In preliminary experiments, the abundance of *Intestinimonas* spp. was significantly greater in the ABX group than in the CON and FMT groups, and genes associated with gametocyte metabolism were markedly altered in the ABX group. These findings suggest that *Intestinimonas* may play a role in inhibiting *E*. *tenella* gametocyte development. To test this hypothesis, we employed *I*. *butyriciproducens* to investigate its specific effects on *E*. *tenella*.

**Fig. 5:**
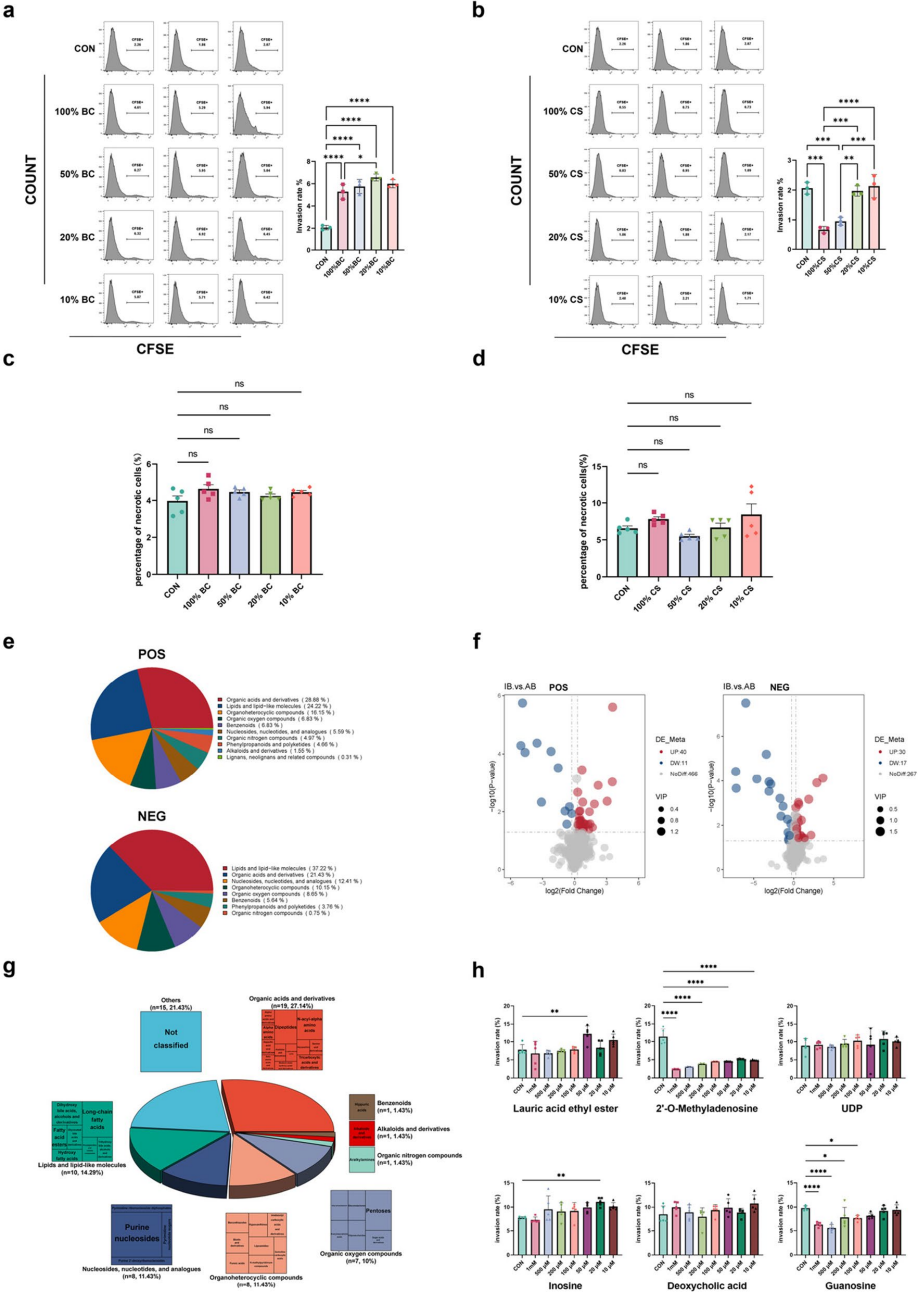
The culture supernatant of*I*. *butyriciproducens* inhibits *E*. *tenella*sporozoite invasion, with 2′-O-methyladenosine identified as the active inhibitory component. **a** Flow cytometry was used to evaluate the effects of BC and **b** CS on *E*. *tenella* sporozoite invasion (*p < 0.05; **p < 0.01; ***p < 0.001; ****p < 0.0001). The results indicate that the culture supernatant of *I*. *butyriciproducens* significantly inhibited *E*. *tenella* sporozoite invasion. The 7-AAD staining method was used to evaluate the direct effects of *I*. *butyriciproducens* cells (**c**) and culture supernatant (**d**) on merozoite mortality. These results indicate that neither the bacterial cells nor the culture supernatant exerted a significant lethal effect on the merozoites. Statistical significance was determined by ANOVA. **e** Nontargeted metabolomics was used to detect the main components in the culture supernatant of *I*. *butyriciproducens*. A total of 517 metabolites were identified in positive ion mode, and 314 metabolites were identified in negative ion mode. **f** Volcano plot of differentially abundant metabolites. In positive ion mode, the abundance of 40 metabolites was significantly increased, whereas the abundance of 11 was significantly decreased; in negative ion mode, the abundance of 30 metabolites was significantly increased, and the abundance of 17 was significantly decreased. **g** The 70 metabolites with significantly increased abundance from both modes were combined for analysis, and a pie chart was generated to illustrate the proportions of each substance type according to classification. **h** Flow cytometry was used to assess the effects of six differentially abundant metabolites on sporozoite invasion. The results indicate that only 2′-O-methyladenosine inhibited sporozoite invasion across all the concentration gradients tested. The results of the metabolite cytotoxicity assays are shown in the supplementary figures

In the initial in vitro experiment, both *I*. *butyriciproducens* cells and their culture supernatant were applied to *E*. *tenella* sporozoites to evaluate their effects on the invasion process. Flow cytometry results demonstrated that BC did not inhibit *E*. *tenella* invasion (Fig. 5a), whereas CS significantly suppressed invasion (Fig. 5b), suggesting that active inhibitory components are likely present in the supernatant. We subsequently coincubated *I*. *butyriciproducens* cells and the culture supernatant with purified merozoites for 12 h and assessed their effects using 7-AAD staining. The results indicated that neither the bacterial cells (Fig. 5c) nor the culture supernatant (Fig. 5d) significantly affected *E*. *tenella* merozoites, confirming that inhibition is specific to sporozoite invasion.

To identify the inhibitory components in the culture supernatant, we analysed the supernatant after culturing *I*. *butyriciproducens* in AB medium for 48 h, using sterile AB medium as a control, and screened for differentially abundant metabolites. In total, 517 metabolites were identified in both positive and negative ion modes (Fig. 5e), with 314 metabolites detected in negative ion mode. Organic acids and their derivatives, as well as lipids and lipid-like molecules, were the most abundant classes, accounting for 53.1% and 58.65% of the metabolites in each mode, respectively. By applying stringent selection criteria, we identified 40 metabolites with increased abundance in positive ion mode and 30 in negative ion mode (Fig. 5f), yielding a total of 70 metabolites with increased abundance. These included 19 organic acids (27.14%), 10 lipids (14.29%), 8 nucleosides and 8 organic heterocyclic compounds (11.43% each), 7 organic oxygen compounds (10%) and smaller proportions of benzenoids, alkaloids, organic nitrogen compounds, and unclassified compounds (Fig. 5g).

To refine the selection of inhibitory components, we excluded irrelevant metabolites and applied stricter screening thresholds (fold change [FC] ≥ 2), yielding 12 differentially abundant metabolites (Table 3). On the basis of the FC, *P* value and VIP rankings, we selected the top five metabolites and ultimately identified six candidate differentially abundant metabolites: lauric acid ethyl ester, 2′-O-methyladenosine, uridine diphosphate (UDP), inosine, deoxycholic acid and guanosine. These metabolites were tested in a DF-1 cell invasion model at various concentrations (1 mM, 500 μM, 200 μM, 100 μM, 50 μM, 20 μM, 10 μM and 0 μM) (Fig. 5h). Prior to the experiments, we evaluated the effects of the metabolites on cell viability and confirmed that, within this concentration gradient, they had no significant effect on cell viability (Supplementary Fig. 4). The results demonstrated that neither UDP nor deoxycholic acid significantly affected sporozoite invasion, whereas 50 μM lauric acid ethyl ester and 20 μM inosine significantly promoted invasion (*p* < 0.01). Guanosine significantly inhibited sporozoite invasion at 1 mM and 500 μM (*p* < 0.0001) and exhibited inhibitory effects at 200 μM and 100 μM (*p* < 0.05). Notably, across all the concentrations tested, 2′-O-methyladenosine significantly inhibited *E*. *tenella* sporozoite invasion (*p* < 0.0001).

**Table 3:** Metabolite differential screening results

Name	FC	P value	VIP
[NEG] Lauric acid ethyl ester	12.59	0.0001	1.52
[POS] 2′-O-Methyladenosine	11.78	0.0000	1.57
[POS] UDP	11.54	0.0009	1.56
[POS] Inosine	8.25	0.0043	1.55
[NEG] Deoxycholic acid	7.43	0.0001	1.51
[NEG] Xanthosine	4.49	0.0277	1.35
[NEG] Guanosine	3.68	0.0007	1.49
[POS] Hypoxanthine	3.61	0.0054	1.50
[NEG] d-Ribose	2.57	0.0367	1.27
[POS] dl-Norvaline	2.54	0.0411	1.28
[NEG] d-Ribose-1-phosphate	2.21	0.0358	1.28
[POS] *N*-Acetylneuraminic acid	2.03	0.0264	1.35

In conclusion, these findings confirm that 2′-O-methyladenosine is the key bioactive component in the *I*. *butyriciproducens* culture supernatant responsible for inhibiting *E*. *tenella* sporozoite invasion.

**Fig. 6:**
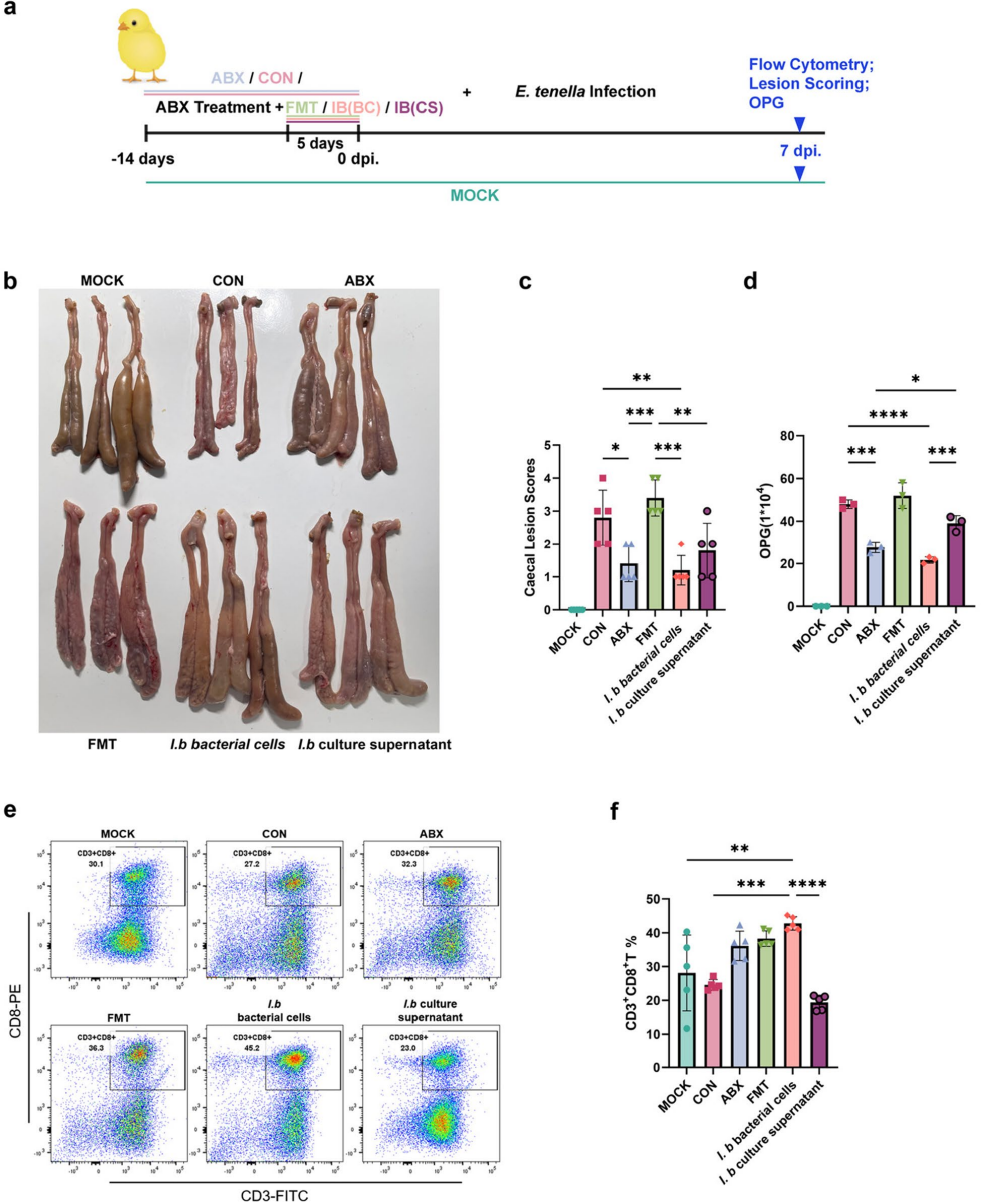
The in vivo anticoccidial effects of *I*. *butyriciproducens* cells are significantly greater than those of the *I*.* butyriciproducens* cell culture supernatant. **a** This experiment included six groups: the MOCK, CON, ABX, FMT, *I*.* butyriciproducens* bacterial cell groups and the *I*. *butyriciproducens* culture supernatant group. AA broilers received ABX treatment starting at 1 day old, and from 10 days old, they were continuously supplemented with *I*. *butyriciproducens* bacterial cells or culture supernatant for 5 days before *E*. *tenella* infection. **b** Representative macroscopic images of chicken caeca and **c** lesion scores from the MOCK, CON, ABX, FMT, *I*.* butyriciproducens* cell and *I*. *butyriciproducens* culture supernatant groups. The results indicate that *I*. *butyriciproducens* cells significantly alleviate caecal lesions induced by *E*. *tenella* (**p < 0.01), whereas its culture supernatant has no significant effect. **d** OPG for each group. *I. butyriciproducens* significantly reduced *E*. *tenella* oocyst shedding (****p < 0.0001), whereas its culture supernatant had no effect. **e** Flow cytometry analysis of CD3^+^CD8^+^ T cell proportions in caecal tonsils across groups at 7 dpi, with statistical significance determined by ANOVA (**f**). The results showed that *I*. *butyriciproducens* effectively increased CD8^+^ T cell proportions in the caecal tonsils, while the culture supernatant had no effect

Following our previous in vitro experiments, which demonstrated that the culture supernatant of *I*. *butyriciproducens* specifically inhibited *E*. *tenella* sporozoite invasion, we further investigated whether this strain and its supernatant confer protective effects against *E*. *tenella* infection in vivo (Fig. 6a). Six experimental groups were established: the MOCK, CON, ABX, FMT, IB and CS groups. AA broilers received ABX treatment starting from day 1, and from day 10, either *I*. *butyriciproducens* cells or their supernatant was administered daily for 5 days prior to infection with *E*. *tenella*.

Representative macroscopic images of caecal tissue collected at 7 dpi demonstrated that compared with the other groups, the IB group exhibited healthier ceca with fewer lesions, whereas the CS group showed no significant protective effect (Fig. 6b). Lesion scoring further revealed that, compared with the MOCK, CON and ABX groups, the IB group had significantly fewer caecal lesions (*p* < 0.01), whereas the CS group exhibited no significant reduction in lesion severity (Fig. 6c). Analysis of OPG counts revealed that oocyst shedding was significantly reduced in the IB group (*p* < 0.0001), corroborating its anti-infection efficacy; however, no significant reduction in oocyst shedding was observed in the CS group (Fig. 6d).

Flow cytometry analysis of CD3^+^CD8^+^ T cell proportions in the caecal tonsils at 7 dpi revealed a marked increase in the proportion of CD8^+^ T cells in the IB group, suggesting an enhanced immune response (Fig. 6e). Statistical analysis confirmed a significant increase in the proportion of CD8^+^ T cells in the IB group (*p* < 0.05) compared to the CON group, whereas that in the CS group did not significantly differ (Fig. 6f).

These findings demonstrate that *I*. *butyriciproducens* cells, but not their culture supernatant, effectively enhance the host immune response and mitigate lesion severity and oocyst shedding induced by *E*. *tenella* infection.

The results from the bacterial cell and culture supernatant treatments revealed a critical distinction: while live *I*. *butyriciproducens* cells conferred significant protection and markedly enhanced CD8^+^ T cell responses in the caecal tonsils, its CS failed to recapitulate these effects, showing no capacity to enhance immune responses.

**Fig. 7:**
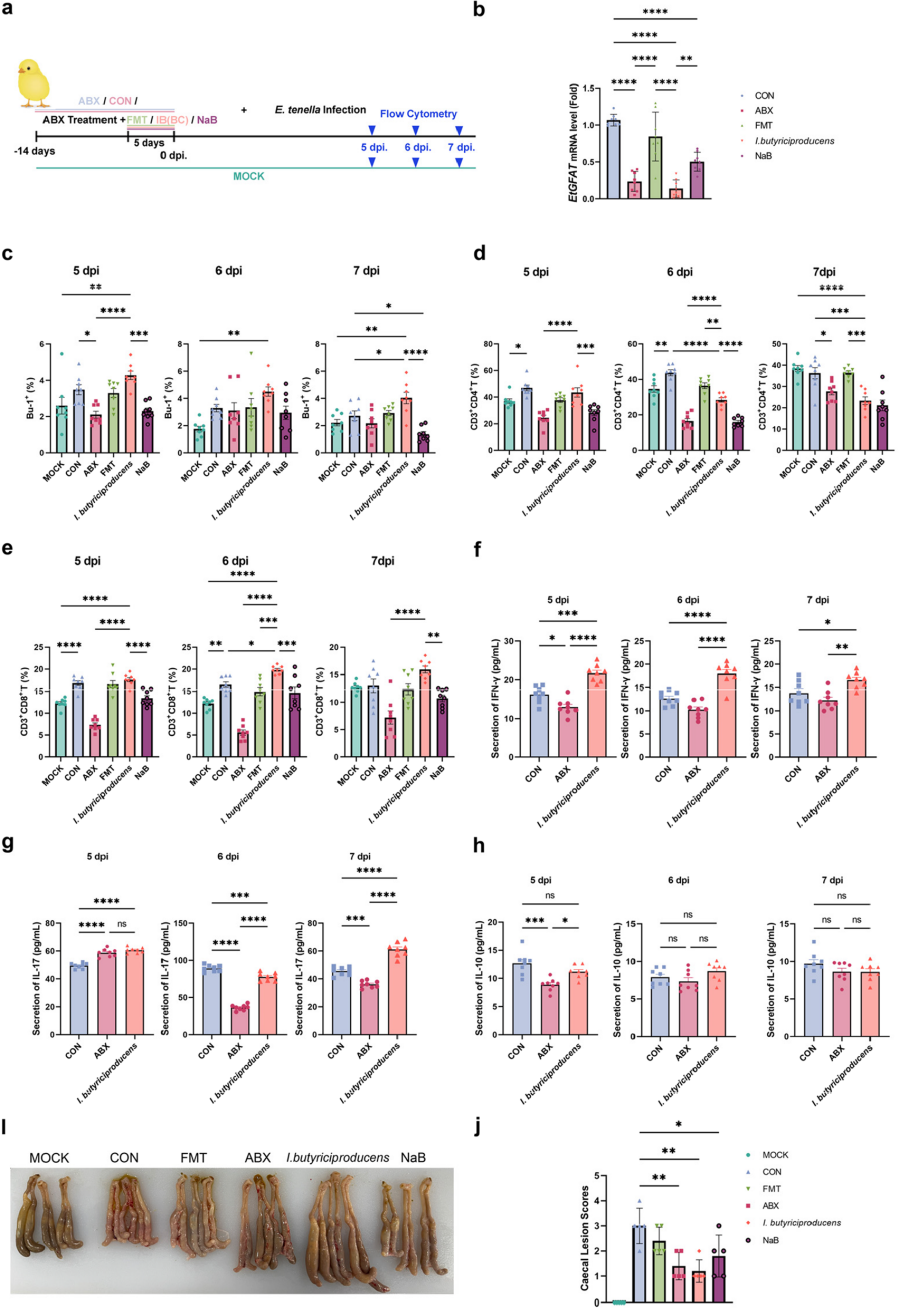
*I*. *butyriciproducens* confers protection against *E*. *tenella* infection by inhibiting the expression of the *EtGFAT* gene and enhancing T cell-mediated immune responses. **a** *I*. *butyriciproducens* colonisation and sodium butyrate (NaB) supplementation experiments. The NaB group is the sodium butyrate drinking group, which is administered simultaneously with *I*. *butyriciproducens*. Sodium butyrate was added to the daily drinking water at a dose of 5 g/L. **b** Expression of the *EtGFAT* gene in the CON, ABX, FMT, *I*. *butyriciproducens*, and NaB groups was measured by qPCR (**p < 0.01, ****p < 0.0001). **c** Percentages of Bu-1⁺ cells in peripheral blood across the MOCK, ABX, CON, FMT, *I*. *butyriciproducens* and NaB groups at 5, 6 and 7 dpi. The error bars represent the standard deviation (*p < 0.05, **p < 0.01, ***p < 0.001, ****p < 0.0001). **d** CD3^+^CD4^+^ T cell percentages and **e** CD3^+^CD8^+^ T cell percentages at 5, 6 and 7 dpi. The bar graphs show the percentage of CD3^+^CD4^+^ T cells in different experimental groups, including the MOCK, CON, ABX, FMT, *I*. *butyriciproducens*, and NaB groups, at 5 dpi (left), 6 dpi (middle) and 7 dpi (right). Data are represented as the mean ± standard deviation, with individual data points shown. Secretion of IFN-γ (**f**), IL-17 (**g**) and IL-10 (**h**) at 5, 6 and 7 dpi. The bar graphs show the secretion levels in different experimental groups, including CON, ABX and *I*. *butyriciproducens*, at 5 dpi (left), 6 dpi (middle) and 7 dpi (right). Data are presented as the mean ± standard deviation, with individual data points shown (*p < 0.05, **p < 0.01, ***p < 0.001, ****p < 0.0001). **i** Representative images of caecal tissues from different treatment groups (MOCK, CON, FMT, ABX, *I*. *butyriciproducens*, NaB). **j** Comparison of caecal lesion scores between different treatment groups (*p < 0.05, **p < 0.01, ***p < 0.001)

This ruled out a primary role for preformed, stable metabolites in the supernatant and instead directed our hypothesis towards the biological activity of the live bacteria in vivo. We reasoned that the efficacy of live *I*. *butyriciproducens* could be mediated through its sustained production of immune-stimulating metabolites within the host microenvironment. Given its well-established role as a prolific butyrate producer, we hypothesised that butyrate might be a key mediator of the observed host-directed immunity. To test this hypothesis directly and to distinguish the effects of this specific metabolite from the complex biological actions of the live bacteria, we established the following experimental groups: MOCK, CON, ABX, FMT, *I*.* butyriciproducens* (IB), and sodium butyrate (NaB) (Fig. 7a).

First, we assessed *EtGFAT* gene expression levels (Fig. 7b). At 6 dpi, the results of the qPCR analysis revealed significantly lower *EtGFAT* expression levels in the ABX and* I*. *butyriciproducens* groups than in the CON group (*p* < 0.0001). Notably, NaB also significantly suppressed *EtGFAT* expression (*p* < 0.0001), indicating that both *I*. *butyriciproducens* and sodium butyrate inhibit *EtGFAT* expression.

To investigate the immune response, we evaluated B and T cell numbers in the peripheral blood from 5 to 7 dpi. The results demonstrated that *I*.* butyriciproducens* significantly increased the proportion of Bu-1^+^ cells at all time points (5 dpi, 6 dpi and 7 dpi). Notably, at 5 dpi and 7 dpi, the proportion of Bu-1^+^ cells in the *I*. *butyriciproducens* group was significantly greater than that in the control groups (MOCK and CON) as well as in the other experimental groups (ABX, FMT and NaB). These findings suggest that *I*. *butyriciproducens* promotes the expansion of Bu-1^+^ cells (Fig. 7c).

Additionally, in the *I*. *butyriciproducens* group, the number of CD4^+^ T cells on days 6 and 7 dpi was significantly lower than that in the CON group (Fig. 7d). In contrast, the number of CD8^+^ T cells in the IB group remained elevated from days 5 to 7 dpi, with the highest levels observed across all the experimental groups (Fig. 7e).

From 5 to 7 dpi, serum levels of IFN-γ, IL-17 and IL-10 were analysed. IFN-γ levels in the IB group were significantly higher than those in the CON and ABX groups throughout this period (Fig. 7f). Analysis of IL-17 levels revealed that at 5 dpi, the CON group exhibited significantly lower IL-17 levels than the ABX and IB groups did. At 6 dpi, the CON group displayed the highest IL-17 levels, which exceeded those in the ABX and IB groups. By 7 dpi, the IB group had higher IL-17 levels than the ABX and CON groups did, with the CON group having significantly higher levels than the ABX group did (Fig. 7g).

Additionally, we evaluated serum levels of secreted IL-10. The results demonstrated that the IL-10 levels in the IB group remained relatively stable from days 5 to 7 dpi, with a significant difference observed only at 5 dpi compared with those in the ABX group. No significant differences in IL-10 secretion were detected between the IB group and the other two groups (Fig. 7h).

At 7 dpi, the caecal lesions were histologically examined (Fig. 7i). The CON group exhibited significant caecal shortening, wall thickening and congestion, which are characteristic of *E*. *tenella* infection. The ABX group displayed milder lesions, while the FMT group showed moderate symptoms. Notably, compared with the NaB group, the IB group had the mildest lesions, which were even less severe (Fig. 7j). Furthermore, relative weight gain, oocyst discharge and the anticoccidial index (ACI) were recorded for each group. The IB group achieved a weight gain rate of 98%, a 42.45% reduction in oocyst discharge and an ACI of 162, demonstrating potent anticoccidial efficacy (Table 4).

**Table 4:** Statistical results of each data item

Group	Average body weight gain (g)	Relative body weight gain rate (%)	Oocyst shedding per gram (× 10^4^)	Oocyst decrease ratio (%)	Lesion value	Survival rate (%)	Anticoccidial Index (ACI)
MOCK	60.20 ± 3.91	100	0	100	0	100	200
CON	29.15 ± 2.88	48.7	37.80 ± 3.35	0	36	100	72.7
FMT	54.33 ± 3.09^***^	90.6	34.20 ± 1.92	8.93	28	100	122.6
IB	58.77 ± 3.58^***^	98	21.60 ± 1.67	42.45	16	100	162
NaB	52.57 ± 2.39^***^	87.7	25.20 ± 2.86	33.05	18	100	149.6

*p < 0.05; **p < 0.01 markers; ***p < 0.001, for the experimental group compared to the CON group

**Fig. 8:**
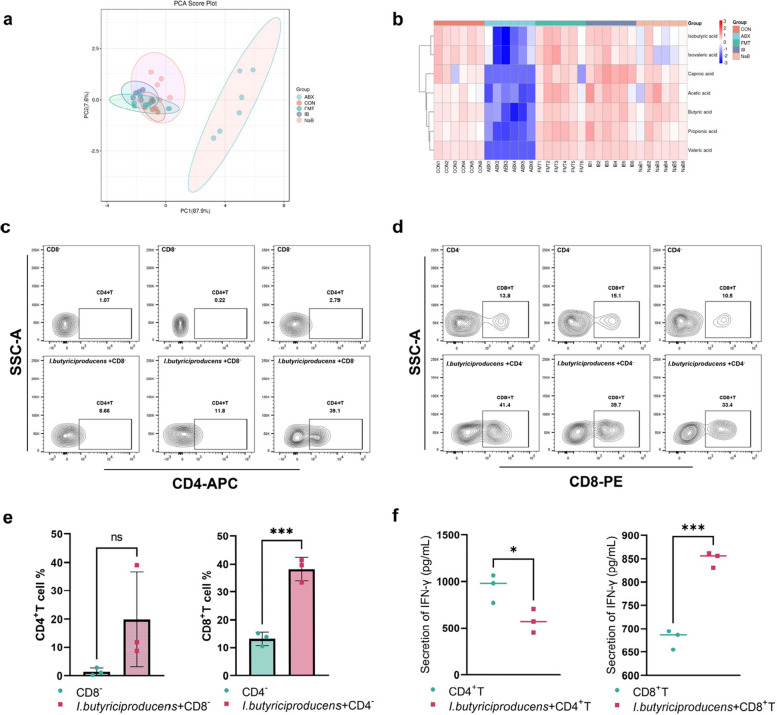
*I*. *butyriciproducens* increases caecal short-chain fatty acid levels during the gametogony stage of *E*. *tenella* and significantly stimulates CD8⁺ T cells to secrete high levels of IFN-γ in vitro. **a** Principal component analysis (PCA) plot illustrating sample clustering from different groups (ABX, CON, FMT, IB, and NaB) based on principal components. **b** Clustered heatmaps illustrating the relative levels of SCFAs across different groups: CON, ABX, FMT, IB and NaB. The colours represent the relative abundance of each SCFA, with red indicating higher levels and blue indicating lower levels. **c** Flow cytometry analysis of CD4⁻ T cell percentages in CD8⁻ cell complexes (comprising CD4⁺ T cells and other antigen-presenting cells) cocultured with *I*. *butyriciproducens*, suggesting that *I*. *butyriciproducens* does not significantly stimulate CD4⁺ T cell proliferation. **d** Flow cytometry analysis of CD8⁺ T cell percentages in CD4⁻ cell complexes cocultured with *I*. *butyriciproducens*, indicating that *I*. *butyriciproducens* significantly stimulates CD8⁺ T cell proliferation in the absence of CD4⁺ cells or within CD4⁻ cell complexes. **e** Analysis of CD4⁺ T cell and CD8⁺ T cell percentages (***p < 0.001). **f** Analysis of IFN-γ secretion by CD4⁻ cell complexes and CD8⁻ T cell complexes cocultured with *I*. *butyriciproducens* (***p < 0.001)

Given that *I*. *butyriciproducens* can synthesise butyrate and potentially convert it into other SCFAs under specific conditions, we measured SCFA levels in the caecum at 6 dpi (Supplementary Fig. 5). PCA revealed distinct clustering of samples in the CON, FMT and IB groups, which were clearly separated from those in the ABX group (Fig. 8a). Heatmap analysis corroborated these findings, demonstrating that the levels of acetic acid, butyric acid, propionic acid, valeric acid and caproic acid were significantly lower in the ABX group than in the other groups (Fig. 8b). Collectively, these findings suggest that the inhibitory effect of *I*. *butyriciproducens* on *E*. *tenella* infection is more likely attributed to its immune-stimulating properties than to the levels of a single type of fatty acid.

To further define the immunological effects of *I*. *butyriciproducens*, we performed flow cytometric sorting on spleen cells from healthy chickens to isolate CD4^−^ and CD8^−^ cell complexes. *I*. *butyriciproducens* was cocultured with CD4^−^ and CD8^−^ cell complexes (Fig. 8c and d), and flow cytometry was used to quantify the numbers of CD4^+^ and CD8^+^ T cells. The results demonstrated that *I*. *butyriciproducens* had no significant effect on the proliferation of CD4^+^ T cells but did significantly stimulate CD8^+^ T cell proliferation (*p* < 0.001; Fig. 8e). IFN-γ levels in the supernatant of the coculture system were also measured (*p* < 0.001; Fig. 8f). These findings indicated that coculturing *I*. *butyriciproducens* with the CD8^−^ complex significantly increased the levels of IFN-γ secreted by CD8^+^ T cells, a response that significantly differed from that of CD8^+^ T cell culture alone. In contrast, *I*. *butyriciproducens* did not significantly stimulate CD4^+^ T cells to secrete high levels of IFN-γ, and the IFN-γ levels in the supernatant were slightly lower than those in the CD4^+^ T cell culture alone. These results suggest that *I*. *butyriciproducens* may regulate the anti-*E*.* tenella* immune response by increasing CD8^+^ T cell activity and promoting IFN-γ secretion by CD8^+^ T cells.

## Discussion

The complex interactions among the gut microbiota, gut parasites and hosts have long been a central focus of research [25–27]. *E*. *tenella* predominantly parasitises the caecum, leading to highly infectious and destructive outcomes. Chickens harbour diverse microbial communities, which profoundly influence their immune and growth performance [28]. Although the epidemiology and life cycle of chicken coccidiosis have been relatively well documented, the precise role of the caecal microbiota in this process—particularly its regulatory mechanisms influencing parasite development and host immunity—remains poorly understood. In this study, we demonstrate that antibiotic-induced dysbiosis specifically impairs gametogony, leading to the accumulation of malformed gametocytes and a significant reduction in oocyst output, suggesting that a competent microbiota is indispensable for the completion of the parasite’s sexual reproductive phase. FMT successfully restored both the microbial community structure and normal parasitic development, underscoring the functional role of the microbiota in this process.

Research on *E*. *tenella* is limited by the limited availability of advanced molecular tools. While studies have developed pseudogerm-free chicken models using antibiotic cocktails [29, 30], none have specifically targeted coccidia. Drawing on methods established for pseudogerm-free mouse models, we developed a pseudogerm-free chicken model. Species diversity analysis confirmed that this antibiotic cocktail significantly altered the caecal microbiota structure, fulfilling our experimental requirements. While the absolute quantification of bacterial load was not performed, the severe reduction in alpha diversity and the radical shift in community structure indicate that our antibiotic regimen likely resulted in a substantial decrease in total caecal microbial abundance, creating a dysbiotic environment that was unsuitable for normal parasite development. Interestingly, ABX treatment led to a relative increase in the abundance of *Intestinimonas* spp., suggesting that this genus may thrive in, or be more resistant to, the altered ecological conditions created by broad-spectrum antibiotics.

The expression pattern of *E*. *tenella* gametocytes resembles that of *Toxoplasma gondii* [31]. RNA sequencing revealed numerous hypothetical proteins, underscoring the limited molecular understanding of the gametocyte stage. Transcriptomic analysis of gametes isolated from different treatment groups revealed that microbiota integrity profoundly influences gene expression in *E*. *tenella*. We identified *EtGFAT* (*Eimeria tenella* glucosamine-fructose-6-phosphate aminotransferase) as a key microbiota-regulated gene. Its expression was significantly downregulated in the ABX group but restored upon FMT. *EtGFAT* is a critical enzyme in the hexosamine biosynthesis pathway (HBP) and is essential for protein glycosylation and nucleotide sugar metabolism [32, 33]. In *Plasmodium berghei*, the homologous gene *PbGFAT* is expressed during macrogamete development [34]. The oocyst wall of *Eimeria* species is rich in glycoproteins [35]. Thus, the microbiota-dependent regulation of *EtGFAT* likely impaired the synthesis of key structural components necessary for macrogamete development and oocyst wall formation, providing a molecular mechanism for the developmental arrest observed in the ABX group.

Random forest regression analysis of the caecal microbiota at the gametogony stage (6 dpi) revealed that *Intestinimonas* spp. were more abundant in the ABX group, with no significant difference between the CON and FMT groups, suggesting the potential role of *Intestinimonas* species as key inhibitors. This was functionally validated using the model strain *I*. *butyriciproducens*. In vitro, its culture supernatant, but not bacterial cells, significantly inhibited sporozoite invasion. Metabolomic screening revealed the nucleoside analogue 2′-O-methyladenosine as a potent inhibitor, likely interfering with the essential purine salvage pathway of the parasite [36, 37]. However, in vivo, CS administration failed to reduce oocyst shedding or lesion scores, while live *I*. *butyriciproducens* cells were highly effective. This discrepancy may be attributed to pharmacokinetic challenges in vivo, where a single metabolite may be rapidly degraded or diluted, whereas live bacteria can continuously produce and deliver a consortium of bioactive molecules within the caecal microenvironment.

Critically, our data strongly support that the primary mechanism of *I*. *butyriciproducens *in vivo is the potentiation of host immunity. Treatment with live *I*. *butyriciproducens*, but not its culture supernatant, significantly increased the population of CD8^+^ T cells in the caecal tonsils and increased systemic levels of IFN-γ. The essentiality of this adaptive immune response was conclusively demonstrated by the complete abolition of the protective efficacy of IB upon T cell inhibition with cyclosporin A (CsA) (Supplementary Fig. 6). This provides direct functional evidence that an intact T cell response is indispensable for the observed anticoccidial effects. This immune-potentiating effect is the most plausible explanation for the superior performance of live bacteria over the culture supernatant or sodium butyrate (NaB) alone. Butyrate, a major product of *I*. *butyriciproducens* [38], contributes to the suppression of *EtGFAT* and has known immunomodulatory properties [39], but it is insufficient to replicate the full protective effect, suggesting that it is one of several synergistic mechanisms employed by the live bacteria. A limitation of this study is the use of the broad T cell inhibitor CsA rather than CD8-specific depletion tools, which are not readily available for the chicken model; future studies employing more targeted strategies, such as anti-IFN-γ blockade, will be valuable to further refine this mechanistic understanding.

In conclusion, our findings elucidate a complex mechanism by which the caecal microbiota, specifically *Intestinimonas*, regulates *E*. *tenella* infection. We propose that dysbiosis disrupts parasitic gametogony partly through the dysregulation of key metabolic genes such as *EtGFAT*. The probiotic bacterium *I*. *butyriciproducens* protects against coccidiosis primarily by modulating the host immune system to elicit a protective IFN-γ-based CD8^+^ T cell response, which is essential for reducing parasite burden and pathology. The production of metabolites such as 2′-O-methyladenosine and butyrate contributes to its overall efficacy. Our study highlights the therapeutic potential of targeting the gut microbiota-immune-parasite axis and highlights *I*. *butyriciproducens* as a promising novel probiotic for controlling avian coccidiosis.

## Conclusions

This study revealed that caecal microbiota integrity critically governs *E*. *tenella* gametogenesis, with antibiotic-induced dysbiosis impairing macrogamete development via downregulation of the glycosylation enzyme *EtGFAT*. FMT restored parasite maturation, pinpointing *Intestinimonas* spp. as key regulators. *I*. *butyriciproducens* exerted dual anticoccidial effects: its metabolite 2′-O-methyladenosine directly inhibited sporozoite invasion in vitro, while bacterial cells suppressed *EtGFAT* expression in vivo, disrupted oocyst wall formation and enhanced IFN-γ-driven CD8^+^ T cell immune responses to reduce oocyst shedding (42.45%) and lesion severity (ACI = 62). These findings reveal microbiota‒parasite crosstalk as a therapeutic target, suggesting that probiotic strategies leveraging *Intestinimonas* to mitigate poultry coccidiosis without relying on conventional drugs are needed.

## Supplementary Information

Supplementary Material 1.

## Data Availability

No datasets were generated or analysed during the current study.

## Abbreviations

ABX: Antibiotic-induced model of caecal dysbiosis
ACI: Anticoccidial index
BC: Bacterial cells of *Intestinimonas butyriciproducens*
CS: Culture supernatant of *Intestinimonas butyriciproducens*
DEGs: Differentially expressed genes
*EtGFAT*: *Eimeria tenella* Glucosamine fructose-6-phosphate aminotransferase
FISH: Fluorescence in situ hybridisation
GO: Gene Ontology
HE: Haematoxylin and eosin staining
HBP: Hexosamine biosynthetic pathway
IB: *Intestinimonas butyriciproducens* Bacterial cell treatment group
KEGG: Kyoto Encyclopedia of Genes and Genomes
MOCK: Uninfected negative control group
NaB: Sodium butyrate treatment group
NMDS: Non-metric multidimensional scaling
OPG: Oocysts per gram of faeces
PCoA: Principal coordinate analysis
SCFAs: Short-chain fatty acid
