# Supplementary material for: Increased caecal Intestinimonas abundance inhibits E. tenella gametogenesis via EtGFAT regulation and alleviates infection through immunity
Source: Microbiome. 2026 Jan 12;14:62. doi: 10.1186/s40168-025-02302-8 (PMC12888579; doi:10.1186/s40168-025-02302-8)
Supplement: Supplementary file 2 — Supplementary Material 1. [file 40168_2025_2302_MOESM1_ESM.pdf]

Supplementary Figure 1: Caecal microbiota diversity analysis between FMT, CON and ABX

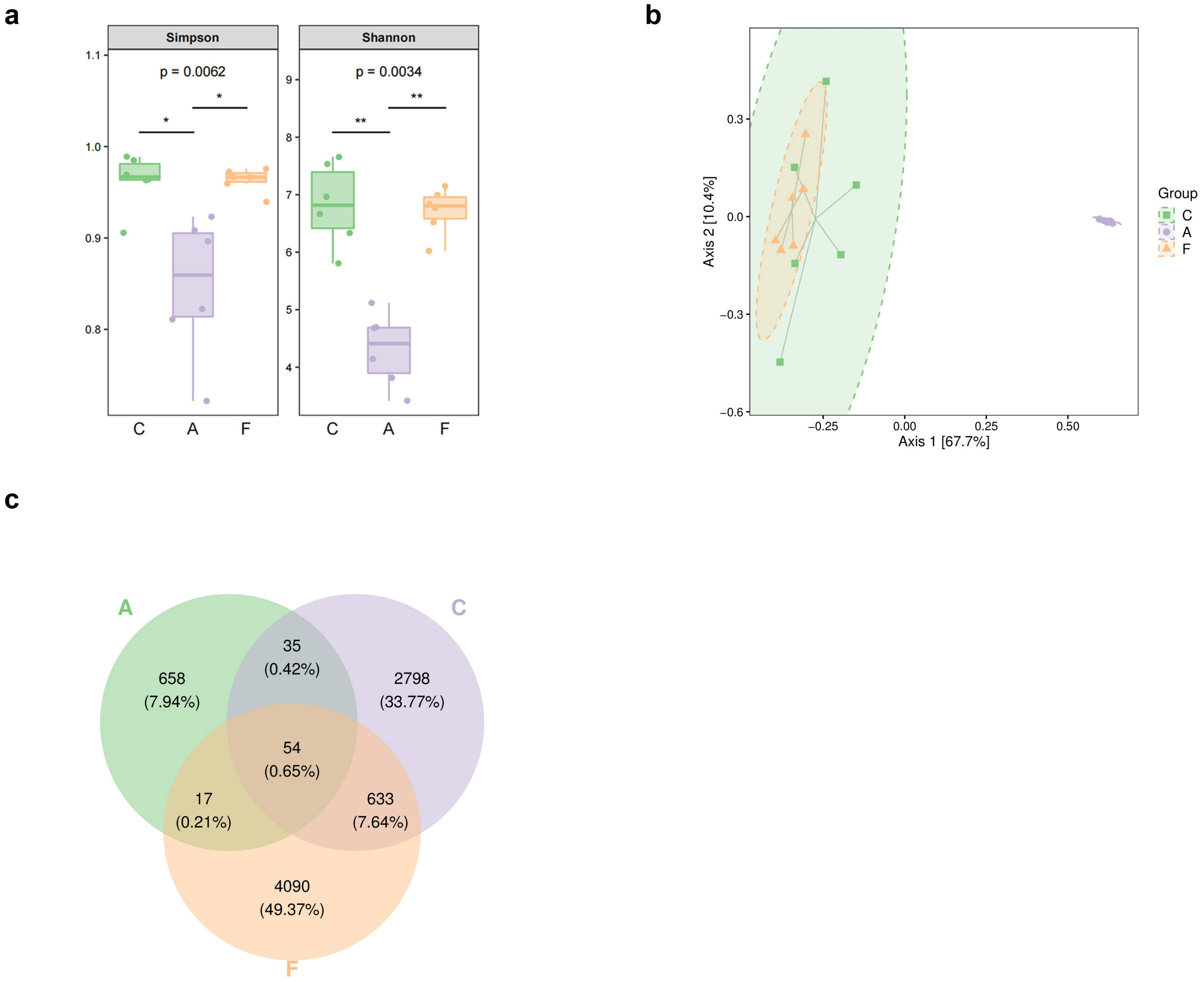

Figure S1. Caecal microbiota diversity analysis between FMT, CON and ABX.

(a) Alpha diversity of the caecal microbiota. Box plots representing the Simpson and Shannon indices show significant differences in microbial diversity among the Control (C), Antibiotic-treated (A), and FMT-treated (F) groups (Simpson index: $p = 0.0062$; Shannon index: $p = 0.0034$, by Kruskal-Wallis test).

(b) Beta diversity of the caecal microbiota. Principal co-ordinates analysis (PCoA) plot based on Bray-Curtis distances reveals distinct clustering of microbial communities among the C, A, and F groups. The first two principal coordinates (PC1 and PC2) explain 67.7% and 10.4% of the total variance, respectively.

(c) Venn diagram illustrates the overlap of caecal microbiota ASVs among the C, A, and F groups.

Supplementary Figure 2: GO enrichment analysis of differential genes (directed acyclic graph)

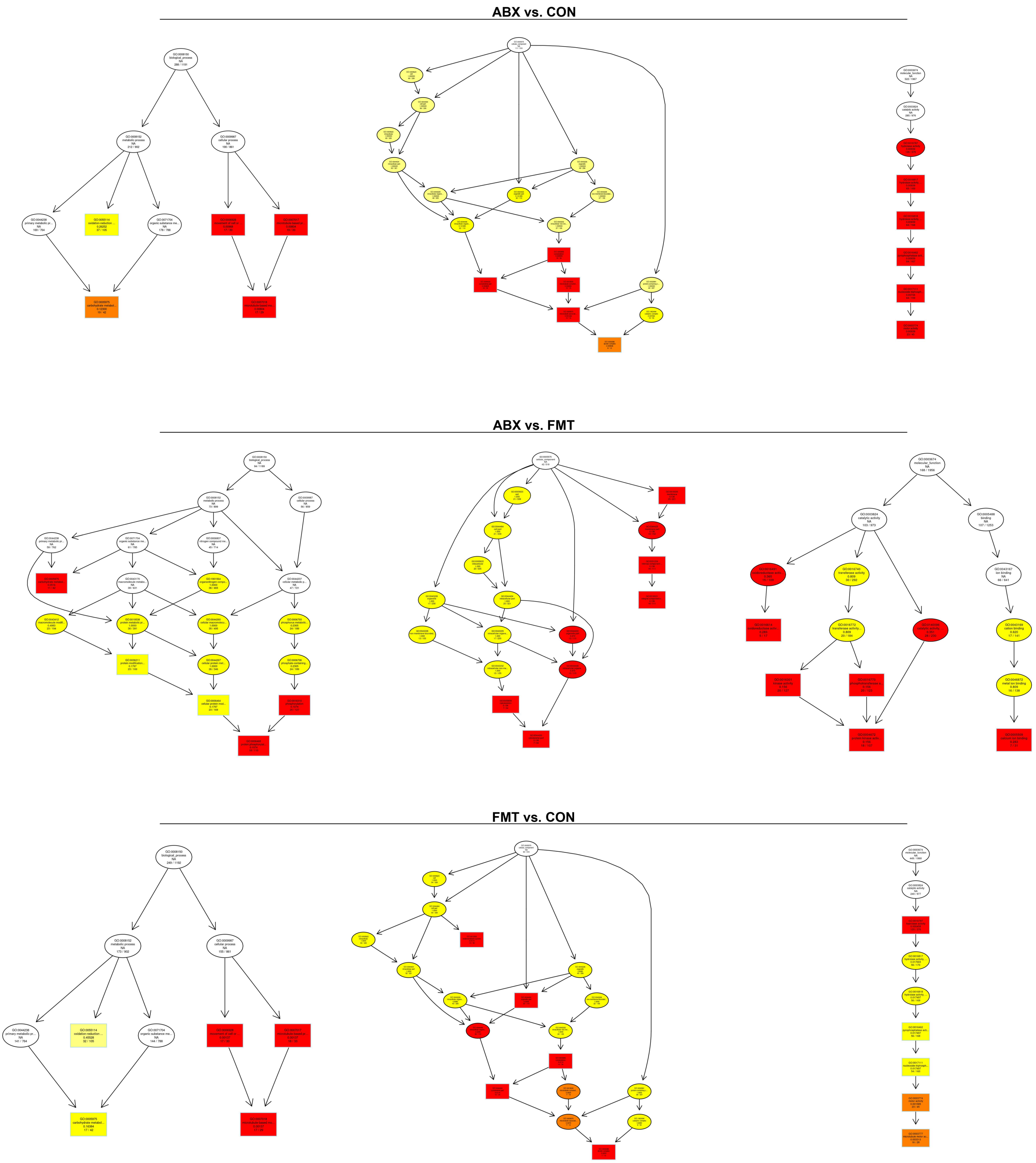

Figure S2. GO enrichment analysis of differential genes (directed acyclic graph) .

Directed acyclic graphs (DAGs) showcase the hierarchical relationships of significantly enriched GO terms from the comparative analyses of (a) ABX vs. CON, (b) ABX vs. FMT, and (c) FMT vs. CON groups. The analysis highlights key pathways influenced by antibiotic-induced dysbiosis and its restoration by FMT.

Supplementary Figure 3: KEGG pathway enrichment of EtGFAT (ETH_00019125)

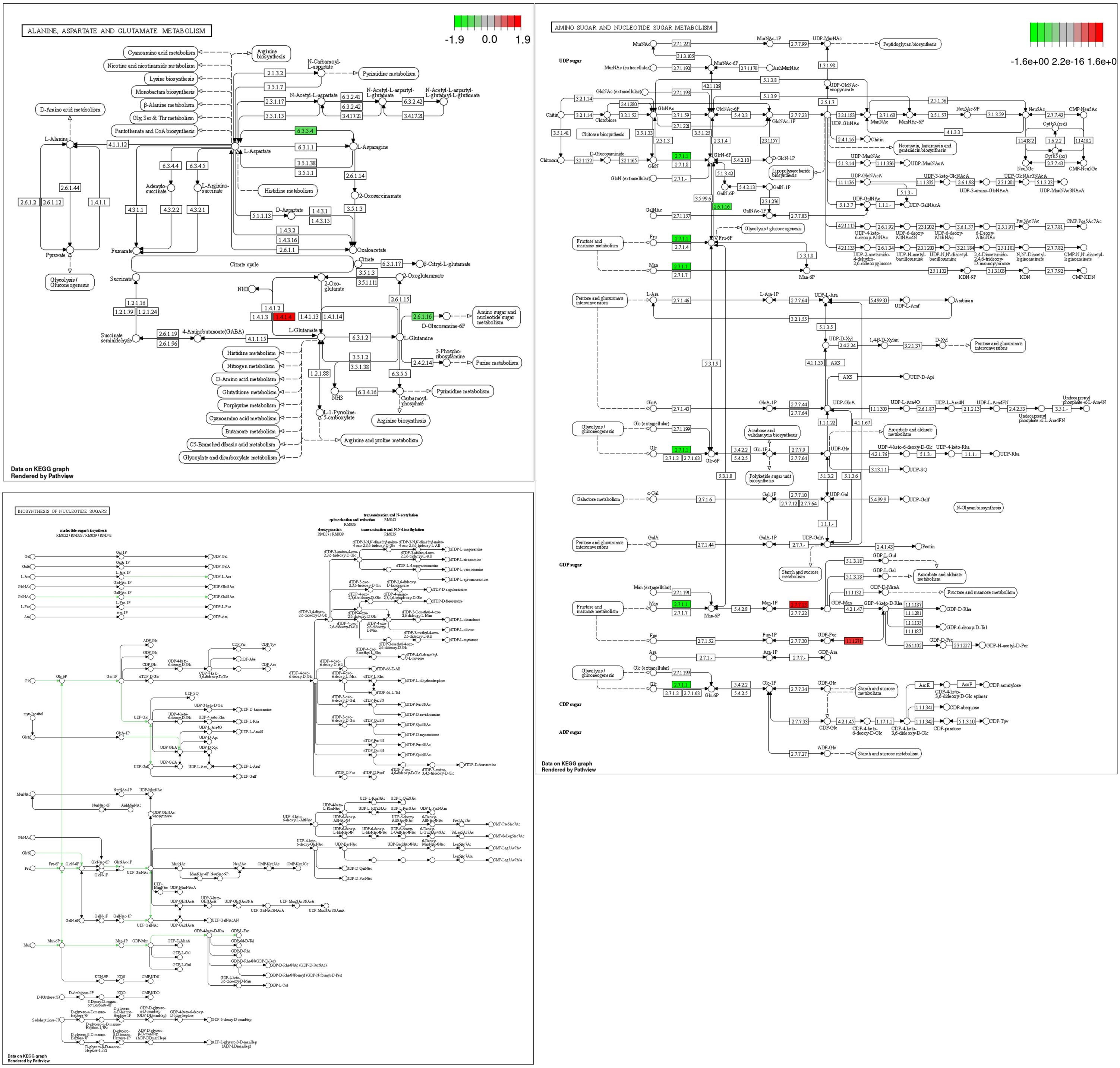

Figure S3. KEGG pathway enrichment of *EtGFAT* (ETH_00019125)

EtGFAT is implicated in amino sugar and nucleotide sugar metabolism.KEGG pathway enrichment analysis reveals that the expression of *EtGFAT* (ETH_00019125) influences the amino sugar and nucleotide sugar metabolism pathway. Nodes are colored based on gene expression changes (red: up-regulated; green: down-regulated), highlighting the central role of EtGFAT in this metabolic process.

Supplementary Figure 4: The effect of six metabolites on DF-1 cell viability (CCK-8).

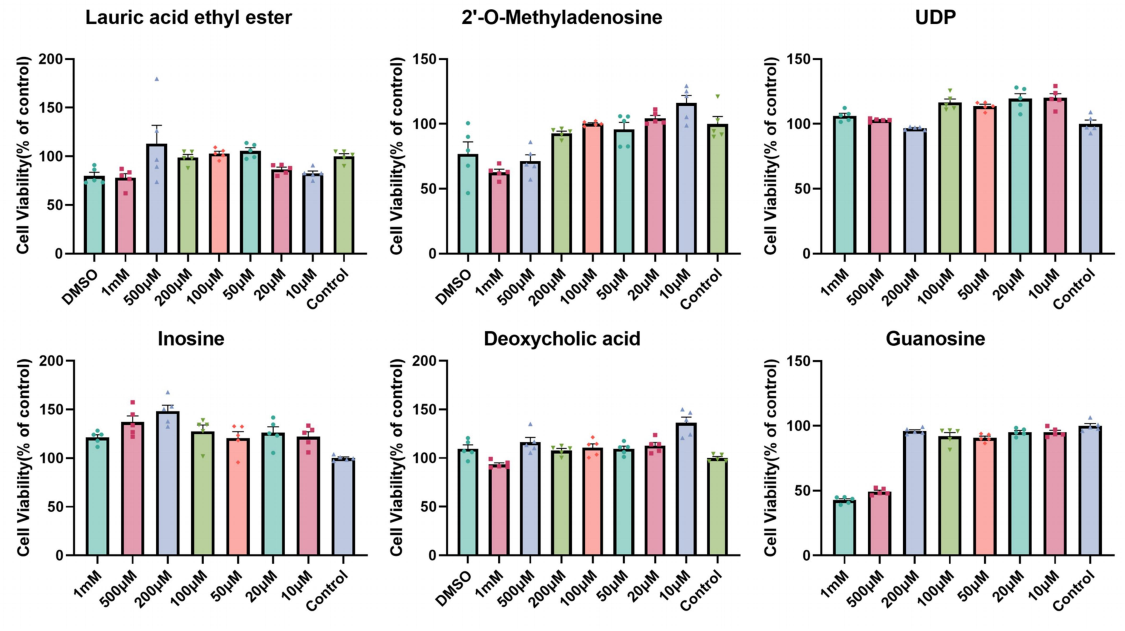

Figure S4. The effect of six metabolites on DF-1 cell viability (CCK-8).

Cell viability was assessed by CCK-8 assay after treatment with varying concentrations (1 mM to 10 μM) of lauric acid ethyl ester, 2'-O-methyladenosine, UDP, inosine, deoxycholic acid, or guanosine. Control groups included DMSO (vehicle) and untreated cells. Data are presented as mean ± SD (n =5).

Supplementary Figure 5: Short-chain fatty acid content and downscaling analysis.

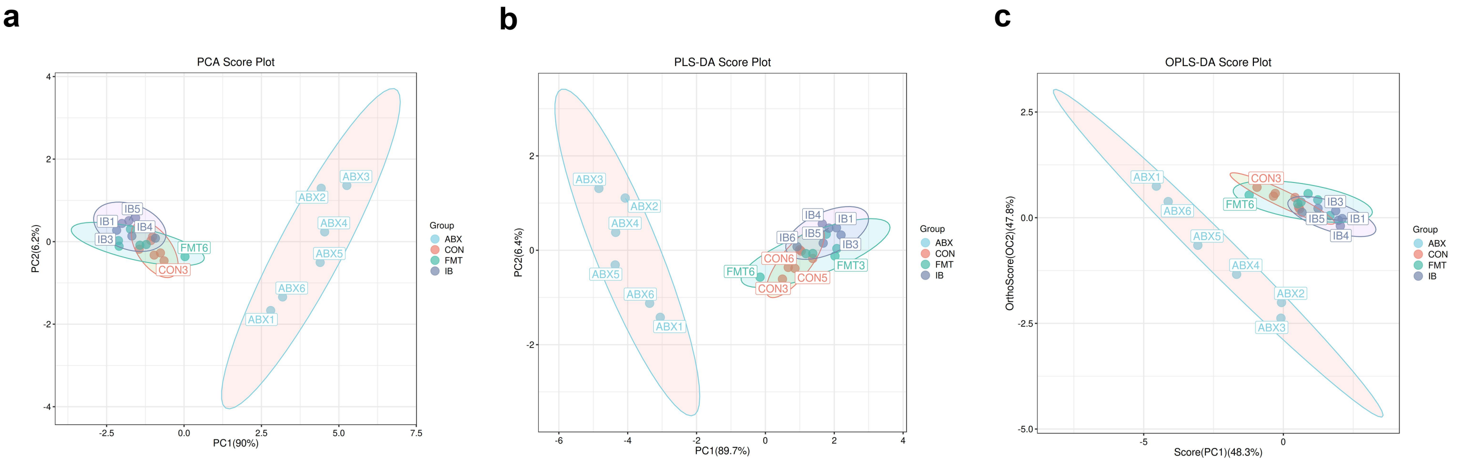

d

Group	Sample volume	Isobutyric acid (μg/g)	Propionic acid (μg/g)	Acetic acid (μg/g)	Butyric acid (μg/g)	Isovaleric acid (μg/g)	Valeric acid (μg/g)	Caproic acid (μg/g)
CON1	50.4mg	79.952	312.3974	1136.8966	166.8141	51.7639	59.2776	0.3831
CON2	51mg	55.8081	236.6635	1131.3827	178.9647	28.7137	34.6364	0.2729
CON3	51.3mg	45.8496	316.445	1045.6326	139.7651	38.6104	30.2729	0.03695
CON4	51.5mg	39.4733	325.9267	1272.9416	263.8536	25.4344	37.0876	0.1305
CON5	50.2mg	58.176	255.5714	925.7553	188.8252	40.0594	33.6645	0.1643
CON6	50.9mg	45.2996	205.6247	829.9348	175.9947	33.4363	33.7518	0.163
ABX1	50.8mg	20.4538	28.0686	215.5539	5.0104	10.7147	ND	ND
ABX2	51mg	0.2064	6.8649	440.4351	5.2894	0.2879	ND	ND
ABX3	50.9mg	0.093	5.9997	307.0527	0.5286	0.1649	ND	ND
ABX4	50.4mg	0.75	8.0604	360.8487	0.1204	0.9942	ND	ND
ABX5	50.7mg	1.4488	9.7332	206.6472	0.2535	1.8848	ND	ND
ABX6	50.1mg	8.1881	20.9744	222.0118	2.4986	8.3556	ND	ND
FMT1	50.6mg	47.6064	465.9243	1131.297	179.1275	37.0566	55.2299	0.1894
FMT2	50.3mg	105.7728	628.6213	1825.4785	360.4	65.916	95.5953	0.3876
FMT3	49.2mg	92.6622	758.2902	1894.1363	386.7266	86.9604	86.5786	0.223
FMT4	50.8mg	56.7744	718.6185	1709.1735	281.8275	33.8472	69.2707	0.3038
FMT5	50.7mg	66.8996	498.1071	1273.9168	200.0824	40.8873	53.3081	0.2191
FMT6	50.6mg	20.8058	325.953	797.6124	113.2456	18.2139	28.3766	0.01479
IB1	49.6mg	69.929	1133.431	2274.4672	328.4995	30.8956	99.8061	0.2378
IB2	49.4mg	74.4571	458.4542	1378.7984	338.1338	36.5242	62.0453	0.44
IB3	50.8mg	75.2483	663.1783	1828.0709	419.2251	57.2288	96.8988	0.6666
IB4	50.7mg	43.7885	468.0667	1605.7192	334.8756	21.0308	73.7708	0.4501
IB5	50mg	48.4612	448.107	1590.0072	559.1371	29.1594	81.586	0.5809
IB6	49.5mg	27.5909	262.841	804.3415	184.9815	28.6879	50.1173	0.4151

Figure S5. Short-chain fatty acid content and downscaling analysis.

Dysbiosis induced by antibiotic treatment alters the caecal short-chain fatty acid (SCFA) profile.

Multivariate statistical analysis reveals distinct SCFA patterns among the Control (CON), Antibiotic-treated (ABX), and Fecal microbiota transplantation (FMT) groups.

(a) Unsupervised PCA score plot showing the natural separation of groups.

(b, c) Supervised PLS-DA and OPLS-DA score plots demonstrating clear separation of SCFA profiles between groups, with the FMT group clustering closer to the CON group, suggesting a partial restoration of the microbial metabolic function.

(d) Quantitative table of caecal SCFA concentrations. The data indicate that antibiotic-induced dysbiosis leads to a significant reduction in key SCFAs like butyrate, which is partially restored by FMT.

Supplementary Figure 6: Cyclosporin A (CsA) immunosuppression abrogates the protective efficacy of *I. butyriciproducens* against *E. tenella* infection.

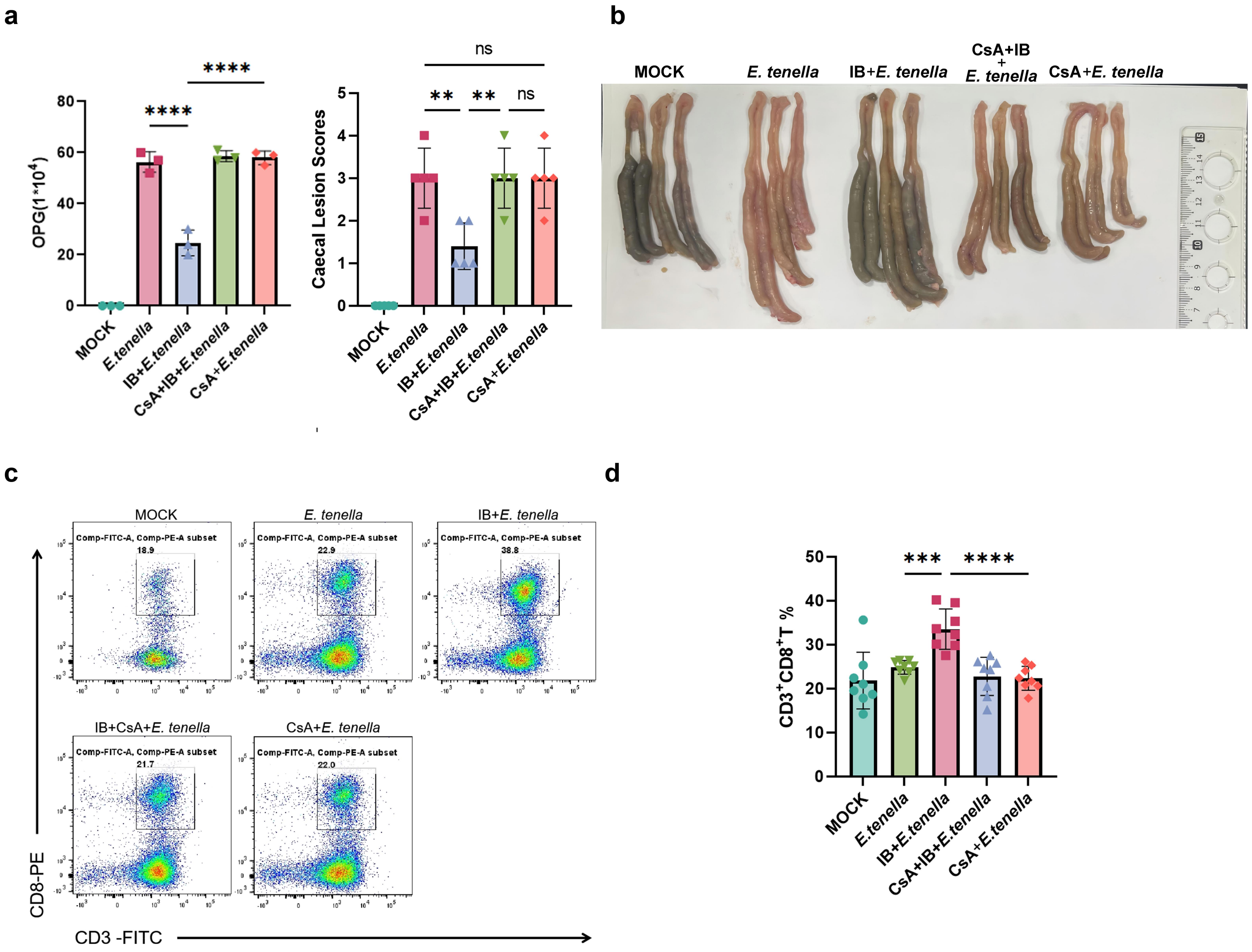

Figure S6. Chickens were treated with Cyclosporin A (CsA, 100 mg/kg, i.m) to inhibit T-cell function. Chicks were challenged with *E. tenella* at 14 days of age. The IB+*E.tenella* group received 1×10⁹ CFU of *I. butyriciproducens* (IB) daily via cloacal inoculation for 5 consecutive days (days 10-14) prior to infection. The CsA+IB+*E.tenella* and CsA+*E.tenella* groups were intraperitoneally injected with cyclosporin A (CsA) at 100 mg/kg every other day from day 1 to day 21 to inhibit T-cell immunity. The MOCK group remained untreated. (a) Oocyst per gram (OPG) of faeces at 7 dpi. (b) Caecal lesion scores at 7 dpi. (c) Representative macroscopic images of caecum. (d) Proportion of $CD3^{+}CD8^{+}$ T cells in caecal tonsils at 7 dpi analysed by flow cytometry. Data are presented as mean ± SD (n=8). Statistical significance was determined by one-way ANOVA with Tukey's post hoc test. The results demonstrate that T-cell immunosuppression completely abolished the anticoccidial efficacy of *I. butyriciproducens* (IB), confirming that an intact T-cell response is essential for its protective effect.
